# Two-Dimensional Graphene Family Material: Assembly, Biocompatibility and Sensors Applications

**DOI:** 10.3390/s19132966

**Published:** 2019-07-05

**Authors:** Xingying Zhang, Ying Wang, Gaoxing Luo, Malcolm Xing

**Affiliations:** 1 Department of Mechanical Engineering, Faculty of Engineering, Department of Biochemistry & Biomedical Genetics, Faculty of Medicine, University of Manitoba Winnipeg, Winnipeg, MB R3T 2N2, Canada; 2 Institute of Burn Research, State Key Laboratory of Trauma, Burn and Combined Injury, Southwest Hospital, Third Military Medical University (Army Medical University), Chongqing 400038, China

**Keywords:** graphene sheet, rGO sheet, GO, aptamer FET sensor, gas sensor, biosensor

## Abstract

Graphene and its chemically exfoliated derivatives—GO and rGO—are the key members of graphene family materials (GFM). The atomically thick crystal structure and the large continuous π conjugate of graphene imparts it with unique electrical, mechanical, optical, thermal, and chemical properties. Although those properties of GO and rGO are compromised, they have better scalability and chemical tunability. All GFMs can be subject to noncovalent modification due to the large basal plane. Besides, they have satisfying biocompatibility. Thus, GFMs are promising materials for biological, chemical and mechanical sensors. The present review summarizes how to incorporate GFMs into different sensing system including fluorescence aptamer-based sensors, field-effect transistors (FET), and electrochemical sensors, as well as, how to covalently and/or non-covalently modify GFMs to achieve various detection purpose. Sensing mechanisms and fabrication strategies that will influence the sensitivity of different sensing system are also reviewed.

## 1. Introduction

Graphene, a two-dimensional (2D) crystal with a hexagonally-arranged carbon atom lattice in the thickness of a single atom, has been found and obtained in 2004 due to the successful mechanical exfoliation of graphite [1]. Since then, many techniques have emerged to exfoliate monolayer graphene including mechanical exfoliation of graphite via Scotch-tape stamping [1], vacuum graphitization caused epitaxial growth of a graphene film on a hydrogen etched SiC wafer [2], chemical vapor deposition (CVD) of hydrocarbons on thin SiO_2_/Si substrates with metal deposition [3], and longitudinal unzipping of multi-walled carbon nanotube (MWCNT) by permanganate oxidation and subsequent chemical reduction [4]. Chemical exfoliation of graphite which starts from the oxidization of graphite to graphite oxide, which was achieved decades ago and substantial methods have been developed since then. However, only the Hummers’ method is still widely used since its high efficiency and satisfying reaction safety [5,6,7,8]. The insertion of oxygen functional groups (OCG) into graphene sheets of graphite impacts the π bonds and causes a stacking interaction between adjacent stacking graphene sheets. Thus, graphite oxide can be subject to further exfoliation through sonication resulting in a single or a few layers graphene oxide (GO), which possess graphene domains, defects, and the inserted OCG existing on the surface and edges of the sheet [9]. The unhybridized P_z_ orbitals of the carbon atoms in graphene not only can form conjugation between two overlapping graphene sheets that leads to a strong interaction between the graphene sheets but also form a large conjugated π system over the graphene plane where electron delocalization (i.e., free transportation of charge carriers) occurs. Therefore, graphene is endowed with excellent electrical and thermal conductivity [10]. The existence of OCG, on the one hand, is beneficial for the GO. On the other hand, OCG breaks the large conjugated system across the GO sheet leading to compromised electrical and thermal properties. Reduction of GO through thermal, chemical, microwave, photochemical, photothermal, or microbial methods gives rise to a class of graphene derivatives that can be described as reduced graphene oxide (rGO) with only a few residual OCG, which restores the supreme properties of graphene [11]. According to the nomenclature recommended by Bianco et al. for graphene and graphene-related materials, GFM—an overarching term—can be used to describe graphene, rGO, GO and other emerging 2D carbon materials in this field [12]. Due to the excellent or/and tunable electrical, mechanical, optical, thermal, and chemical properties possessed by GFM, GFM can be applied in fields such as liquid and gas separation [13], biosensors [14], actuators [15], electronics [16,17], and energy storage and conversion [18]. GFMs are all derived from single or a few layers of 2D hexagonally-arranged carbon atom lattices but will be assembled into macroscopic structures at different dimensional levels while being recruited in sensors. GFMs can play a role in sensors as 2D GFM sheet platforms in fluorescence aptasensors [19,20], nanofillers in composite materials [19,20], building blocks for 2D large-scale films, and interconnected flakes in three-dimensional (3D) aerogels [3,21,22]. Thus, GFMs could be found either as free microscopic sheets or as constituents of a large macroscopic structure (including 2D [23] and 3D [24]), when they are incorporated into sensors. Currently, there are many reviews focusing on the function of GFM-based sensors but messing the two states of GFMs up. Accordingly, these reviews are not informative enough in terms of fabrication details and variety and cannot offer a followable guideline for GFM sensor preparation [13,25,26,27]. The present review summarizes and discusses the state of the art fabrication techniques of incorporating free sheets of GO, rGO, and graphene, which are key members in GFM, into a variety of sensors that can achieve different functions.

## 2. Property of GFM

### 2.1. Electrical, Mechanical, Optical, Thermal, and Chemical

A carbon atom in a graphene lattice has three hybridized sp^2^ orbitals, which are derived from 2S, 2P_x_, and 2P_y_ orbitals of the carbon atom, and each hybridized orbital forms a σ bond with an adjacent atom. Each carbon atom in graphene also has an unhybridized P_z_ orbital perpendicular to graphene, which overlaps with the P_z_ orbital of an adjacent atom and forms a π bond. The π orbital disperses, and as a result, the bonding π band, which corresponds to the lower energy valence band and the anti-bonding π band, which corresponds to the higher energy conduction band, is formed. The gap between the two energy bands closes at Dirac points forming a Dirac cone approximation. Thus, the charge that graphene carries follows a linear dispersion relation and behaves like massless relativistic particles with an energy-independent velocity *v*_F_ of 300 times less than the speed of light *c* [28]. Accordingly, single-layer graphene exhibits extraordinary electrical properties [29]. The electrical conductivity of graphene is 10^4^ S/m, which is five orders of magnitude larger than that of GO [30], and the conductivity of free-standing highly reduced graphene oxide paper ranges from 200 S/m to 35,100 S/m depending on the synthesis process and drying process [31,32]. Graphene also possesses supreme thermal conductivity [33], optical [34], and mechanical stiffness [35]. Young’s modulus of monolayer graphene can reach 1 TPa [35], which is two times higher than monolayer graphene oxide. The thermal conductivity of GFM declines with the decrease of the carbon to oxygen ratio [36,37]. Monolayer graphene shows a 97.7% transparency [38], however, the optical transmittance of rGO and GO is expected to be low because of OCG [39]. Zig-zag and armchair are the two edges observed in graphene of which the chemical reactivity is different from the inert basal plane as σ bonds with adjacent carbon atoms and π conjugation networks break while forming the edges [40]. Thus, functionalization and tailoring of the edges can be achieved by organic chemistry without causing severe changes to the basal plane’s aromatic structure [41]. Defects in the basal plane of graphene can be viewed as edges as they also form the termination of the conjugated network [42]. Thus, they are expected to have increased local reactivity and properties when bonded with hydroxyl, carboxyl, or other active groups [43]. Besides the covalent modifications on the edges and defects, the π conjugation network on the graphene basal plane facilitates non-covalent modification through π–π stacking interactions [41]. Though most properties of graphene discussed above are superior, GO and rGO have their merits of facile fabrication and high scalability. Besides, OCG on a graphene sheet is reactive to substantial chemical species and provides more sites for surface modification to develop functionalized GFMs [9]. Additionally, it was reported that nano-GO, that possessed lateral dimensions down to <10 nm, exhibited a unique photoluminescence spectrum ranging from near-infrared (NIR) to visible light, which can be applied in cellular imaging with little background [44]. Upon excitation with light at a wavelength of 400 nm, a homogeneously dispersed GO solution emits a broad band fluorescence, which peaks at 570 nm.

### 2.2. Biological

#### 2.2.1. Interaction with Biomolecules

Graphene family materials show biological affinity to DNA and RNA [45,46,47,48]. Based on the studies about the adsorption of DNA nucleobases onto graphene, the π-stacking effect, hydrogen bond, hydrophobic interactions and strong van der Waals interactions are considered as the driving forces [49,50,51,52]. Single-stranded DNA (ssDNA) was found to be effectively protected from enzymatic digestion by interacting with sulfuric and nitric acid functionalized graphene as the interaction produces a steric hindrance effect to prevent DNase I binding [52]. GO exhibits preferential affinity to ssDNA compared to double-stranded DNA (dsDNA) [25]. The hydrophobic forces and π–π stacking induced binding of GO to nucleobases of dsDNA need to overcome the electrostatic repulsion between negatively charged GO and polyanionic DNA. The repulsion is usually weakened by the presence of a high ionic strength and a low pH. However, binding of GO to ssDNA, where the nucleobases are not protected by the outer negatively charged phosphate groups, is not impacted by electrostatic repulsion, and thus, is preferred. The adsorption affinity of DNA to rGO is higher than GO [53]. As rGO has broader regions for π–π stacking and less negatively charged carboxyl groups, adsorption of DNA on rGO is faster than GO; moreover, the adsorption of DNA on rGO as a result of pH, urea, temperature, and organic solvent is less likely to happen.

Interaction of GO nanosheets (nS) with other biomolecules has also been investigated. Amino acids either carrying an aromatic group or a positively charged side chain have the potential to bind with GO [54]. Lysine (Lys), histidine (His), arginine (Arg), tryptophan (Trp), tyrosine (Tyr), and phenylalanine (Phe) were used to study the interaction with amino acid and GO. These amino acids could be listed as Phe < Try < Trp < Lys < His which were ranked according to binding strength with GO. The positively charged peptide can bind with GO through hydrophobic interactions, π–π interactions and electrostatic interactions. 

#### 2.2.2. Antibacterial Activity

The antibacterial activity of GFM towards various kinds of bacteria has been studied. According to Liu et al., almost all the members of GFM showed antibacterial activity against Gram-negative *Escherichia coli* species and a three-step anti-microbial mechanism that has been applied to carbon nanotubes could be also used to explain the antibacterial activity of GFM [55]. The first step is a bacterium-GFM through direct contact that resulted from bacterial deposition onto sheet GFM. The second step is an intimate interaction of GFM with bacteria that would result in disruptive membrane stress since GFM has a hydrophobicity related tendency to partition into the cell membrane. The third step includes the disruption of a specific microbial process via superoxide anion-independent oxidation stress. The authors also made comparisons of the antibacterial activity among GFM dispersions of the same concentration. The GO dispersion with the advantage of forming a stable dispersion with small nS due to the insertion of OCG such as carboxyl, hydroxyl, and epoxy groups exhibits the strongest antibacterial activity and is sequentially followed by rGO, which would induce stronger oxidative stress than graphite and graphite oxide. The toxicity of rGO and GO against the Gram-positive *Staphylococcus aureus* has been reported [56]. Meanwhile, the authors point out that *S. aureus* bacteria are less resistant to cellular membrane damage caused by GFM for the lack of an outer membrane. The toxicity of GO against *Shewanella* species has also been studied and has been reported that GO lacks toxicity against the species but could be reduced by the species [57]. Quaternary ammonium salts (QAS) are cationic biocides that are conventionally used to functionalize chitosan to enhance antibacterial activities [58,59]. The antibacterial activity of QAS is also believed to derive from interaction with the cytoplasmic membrane of microbes that disturbs membrane permeability since QAS can form strong electrostatic interactions with the negatively charged bacterial surface [59]. Our group developed a GO-QAS nanocomposite that shows synergistic antibacterial activity including membrane perturbation and oxidative stress [60]. QAS can also act as a surfactant to improve the polydispersity of GO. Moreover, the composite presents amazing biocompatibility both in vivo and in vitro, which makes it a prospective material for wound dressing and antifouling coatings. To synthesize the composite, quaternized monomers were polymerized through reversible addition-fragmentation chain (RAFT) agent assisted polymerization. Amine groups were grafted to GO via salinization using (3-aminopropyl) trimethoxysilane (APTMS). Then, the QAS polymer was grafted to the amine groups on GO via EDC/NHS intermediated coupling. 

#### 2.2.3. Cytotoxicity

The in vitro cytotoxicity of dispersed GFMs in various types of human cells including fibroblasts [61], alveolar basal epithelial cells (A549 cell line) [62,63], skin fibroblasts and red blood cells [64], THP-1 macrophages [65], as well as neuronal cells [66], has been studied. Most GFMs show a dose-dependent cytotoxic effect. GO dispersion with a dose lower than 20 μg/mL imposed little toxicity on fibroblast cells. However, cytotoxic behavior including a decrease in the adhesive ability of the cell, triggering cell apoptosis, internalized by lysosomes, mitochondrion, and the nucleus would be observed if the dose of the GO dispersion was increased to 50 μg/mL [61]. In contrast, GO showed a small toxicity against the A549 cell line, inducing a slight decrease in cell viability and oxidative stress [63]. These effects were not only dose-dependent but also size related. Large and medium GO sheets had little influence on the viability of A459 cells, which retained an 80% viability rate. Although the GO concentration reached 200 μg/mL, a small GO would cause a more severe loss of viability at the same concentration. Exposure to GO dispersion, even at a low concentration, would impart oxidative stress to A549 cells and induce an increase in reactive oxygen species (ROS) level. Small GOs also elicited more serious effects than large and medium GOs. A mild concentration-dependent cytotoxicity of GO against A549 cells was demonstrated by Hu et al. and the toxicity was believed to derive from a direct interaction between the cell membrane and GO, which induced physical damage [62]. Similar to nanoparticles (NP), GFM has la arge surface-mass ratio and a nano size. Thus, it can interact with proteins [67,68]. Proteins can easily absorb on NPs and form an NP–protein complex called a corona. The surface bio-reactivity of NPs will be modified by the corona. It was found that the protein corona on the surface of GO, which was formed by fetal bovine serum (FBS), could greatly alleviate the cytotoxicity of GO at a concentration of 10%. Few-layer graphene sheets synthesized via the radio frequency catalytic CVD technique were found to impose concentration-dependent cytotoxicity on neuronal P12 cells [66]. Several factors of P12 cells were measured upon exposure to graphene sheets at different concentration: metabolic activity, ROS generation, LDH release, which is a hallmark of necrosis and membrane damage, and caspase 3 activity, which is an apoptosis marker. Graphene sheets at a lower dose (0.01 μg/mL) were found to have no effect on those factors, suggesting that graphene sheets at low concentration can be theoretically used for neural system biomedical applications. Whereas, graphene sheets of higher does would elicit oxidative stress and apoptosis-mediated cytotoxicity. The cytotoxicity of graphene sheets in P12 cells was compared with carbon nanotubes. It was found that the morphology of carbonaceous nanomaterials determined how they would interact with cells. According to the comparison of the cytotoxicity of GO and graphene in erythrocytes made by Liao et al., GO had higher dose-dependent hemolytic activity due to a large amount of contact between the cell and the negatively charged OCG on the GO surface. It would have intense electrostatic interaction with the positively charged phosphatidylcholine lipids on red blood cells (RBCs). Besides, better exfoliation and better particle size of GO would result in an even higher hemolytic activity. However, graphene, which tends to form aqueous aggregation and induce hemagglutination surrounding graphene sheet aggregates showed a lower dose-dependent hemolytic activity [64]. Similarly, coating GO sheets with chitosan can significantly suppress hemolysis because the pH-dependent solubility of chitosan makes it form aggregates with GO at pH ~7.4 [69]. The aggregate can mask GO and decrease the electrostatic interaction area of GO. On the other hand, graphene sheets were observed to be more toxic on human skin fibroblast cells than GO, which is likely due to the impaired nutrient adsorption and growth of human skin fibroblasts caused by faster sedimentation and more compact aggregation formation of graphene sheets. Interaction of macrophages with few-layer (1–10 layers) graphene nanoplatelets (GP) was studied by treating with THP-1, which is a differentiated monocytic cell with GP. It was observed that GP could only be partly phagocytosed by THP-1 cells, indicating frustrated phagocytosis [65]. GP also showed a dose-dependent effect on the membrane integrity and ROS level of THP-1 cells. On the other hand, GFM plays an important role in cancer therapy because of its ability to triggering a large generation of ROS under the activation of light source cancer therapy [70,71].

Overall, GMF shows dose-dependent cytotoxicity in various cells, and the cytotoxicity can be reduced by coating GFM with some biocompatible molecules, forming aggregates, and being fabricated into films. The biocompatibility of GFM suggests the potential of being ideal material for biosensors.

## 3. Fluorescence Aptamer/GFM Sensors 

**Figure 1:**
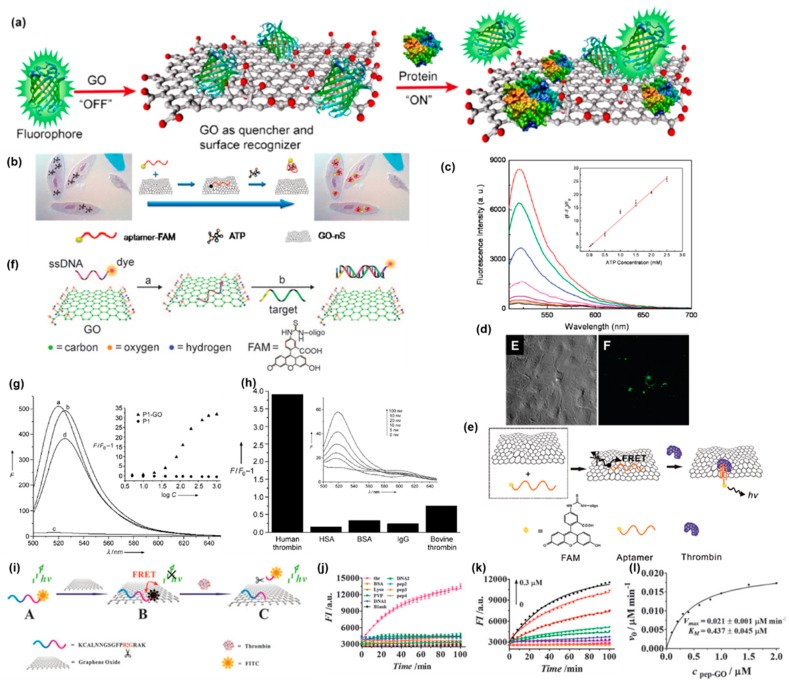
(**a**) Motif and fabrication of the (Graphene oxide) GO nS-based sensor assay. GO adsorption would cause a quench-off of the fluorescence. Releasing of quenched fluorophore due to analyte molecule binding restores the fluorescence. (**b**) Aptamer/GO Nanosheet (nS) nano complex achieves in situ molecular sensing in living cells. (**c**) Quenching of the Adenosine triphosphate (ATP) aptamer- carboxyfluorescein (FAM) because of the GO nS (bottom black line). Fluorescence restoration caused by mixing the aptamer-FAM/GO nS with ATP (from bottom to top). Insert: Response of fluorescence intensity to ATP concentration. (**d**) Images showing the cellular uptake of aptamer-FAM/GO nS by JB6 cells. (**e**) Sodium dodecylbenzene sulfonate (SDBS)-graphene aptasensor and detection mechanism. (**f**) Fluorescence restoration of the ssDNA-FAM/GO complex due to target binding. (**g**) Fluorescence spectra (excited at 480 nm) of a. P1 (50 nM) in Tris-HCl buffer; b. P1+HIV1 (300 nM); c. P1-GO complex; d. P1-GO complex + MHIV1 (300 nM). Insert shows F/F_0_-1of P1 and P1-GO at different concentrations (c) on a logarithmic scale. (**h**) The response of the fluorescence (F/F_0_-1) of the dye-labeled aptamer-GO to binding different proteins. Insert: Response of fluorescence spectra to human thrombin at different concentrations. (**i**) A sensing probe composed of the peptide 1-GO bioconjugate to monitor the proteolytic activity of thrombin. (**j**) Fluorescence spectra of the peptide1-GO complex exposed to different targets. (**k**) Thrombin proteolytic monitoring assay performed on thrombin at different concentrations. (**l**) Protease activity analysis. Reprinted with permission from References [54,73,74,75,77].

As mentioned above, GFM has a strong affinity and specificity for biomolecules and chemically reactive OCG offers active sites for covalent functionalization and tailorable cytotoxicity. Therefore, it shows potential to be used for the design of biosensors. An aptamer is an artificial oligonucleotide or peptide that can bind to various molecules, including protein, with a high affinity and specificity for a given target [72]. GFM can bind with an aptamer complex due to the unique ability of GFM for DNA and peptide adsorbing. Besides, graphene has substantial π system and a continuum of electronic excitation is possible in graphene because of the Dirac cone approximation. Thus, GFM facilitates a rapid resonance energy transfer (RET) between a dye molecule and GFM, which makes GFM an effective fluorescence quencher. The fluorescence of the dye-aptamer complex is quenched immediately as soon as GO has formed the self-assembled bioconjugate with the complex due to the strong and fast fluorescence resonance energy transfer (FRET) from dye molecule to the GO nS. Besides, GFM is not only an effective transporter and quencher for aptamer-fluorophore but also protects the aptamer from being cleaved by an enzyme during delivery. The binding of a specific biomolecule to the dye-aptamer-graphene conjugate would then result in the recovery of fluorescence based on various mechanisms (Figure 1a), which offer a guideline for the design of GFM-based aptasensors [73,74,75].

GO nS, by the virtue of a strong interaction with DNA molecules, were employed by Wang et al. as a vehicle to carry the aptamer of adenosine triphosphate (ATP) in the design of their biosensor and real-time biosensing platform. Their design achieved in situ molecular sensing in living cell systems (Figure 1b) [73]. Fluorophore carboxyfluorescein (FAM) was used to label the ATP. The formation of the aptamer-FAM/GO nS complex with GO would quench the FAM fluorophore. The aptamer-FAM/GO nS complex can be delivered into living cells after being incubated with cells. Aptamer-FAM can bind the target ATP in the cells and form a duplex configuration and adsorption of ATP aptamer-FAM on GO nS is thus weakened, which leads to the release of ATP/aptamer-FAM. As a result, extraordinary fluorescence recovery was viewed in the ATP sites of living cells (Figure 1d). The aptamer-FAM/GO nS can selectively detect ATP in vitro and can respond to a wide range of ATP concentrations. Besides, the relative fluorescence intensity increased linearly with the increase of ATP concentration (Figure 1c). Chang et al. adopted a similar strategy to develop a graphene-based biosensing platform for thrombin detection (Figure 1e) [74]. The aptamer used in their work was an oligonucleotide that will form a quadruplex-thrombin complex upon binding to thrombin, resulting in a conformational change. Accordingly, binding between quadruplex-thrombin complexes and the graphene surface is weakened, increasing the distance between FAM and the graphene surface. The fluorescence can thus be recovered. In their work, GO was dispersed with sodium dodecylbenzene sulfonate (SDBS) to increase the biocompatibility and dispersibility. Thrombin, as a serine protease, is vital in the regulation of tumor growth, metastasis, and angiogenesis [76]. Thrombin plays an important role in angiogenesis since it can induce the formation of fibrin through cleavage of an Arg-Gly bond in fibrinogen. Based on this concept, Zhang et al. synthesized a peptide (peptide 1) that can recognize thrombin due to an enzyme-sensitive core domain (dPhe-Pro-Arg-Gly) and can adsorb on GO to replace the oligonucleotide aptamer on conventional GO-based biosensors (Figure 1i) [54]. The design realizes real-time monitoring of protease activity and is reported to be the first GO-based biosensing platform that does not rely on an ssDNA probe. In their work, the peptide1, which can be proteolyzed by thrombin with the selective cleavage at the Arg-Gly bond, was labeled by Fluorescein isothiocyanate FITC and was conjugated onto the GO surface. The cleaved segment then detached from the GO surface resulting in fluorescence recovery. Therefore, the FITC-peptide1/GO system enabled real-time monitoring of thrombin activity as a protease. The FITC-peptide 1/GO system exhibited a strong selectivity towards thrombin and a high sensitivity to thrombin at different concentrations (Figure 1j,k). To study the protease activity, the real-time fluorescence responses of the FITC-peptide 1/GO complex at different concentrations in the presence of thrombin (300 nM) were recorded and the initial velocity ($\nu_{0}$) of protease hydrolysis was then obtained. $\nu_{0}$ was plotted against the concentration of the peptide-GO complex and the data were fit to the Michaelis-Menten equation through a non-linear least squares regression. Parameters including maximum proteolytic velocity ($V_{max}$), the Michaelis constant ($K_{M}$), and turnover number ($k_{cat}$) were thus determined (Figure 1l).

The potential of water-soluble GO as a platform to sensitively and selectively detect ssDNA and thrombin was demonstrated by Lu et al. (Figure 1f) [77]. As is discussed before, dsDNA shows a weak affinity to GO. Binding the complementary target sequence to the FAM-labeled ssDNA that anchors to GO through noncovalent binding would release the FAM-labeled DNA from GO or increase the distance between GO FAM and GO, which would result in the restoration dye fluorescence. To enable the GO platform to detect DNA, a dye-labeled ssDNA oligonucleotide sequence P1 was used as an aptamer that would adsorb to the surface of GO and form a P1-GO complex probe. HIV1 and MHIV1 are the complementary target sequences and the single-base mismatch sequence for the target DNA respectively and they were used to determine the selectivity of the GO/aptamer-based sensing platform. The relative fluorescence intensity (F/F_0_) of P1-GO towards 150 nM MHIV1 is about 47.1% of that towards 150 nM HIV1. This platform displays a high specificity to the DNA-sequence signal in contrast to the linear DNA probe, which is not able to distinguish single-base mismatch targets. The successful detection of HIV1 over a range of concentrations from 1 nM to 1 μM is shown in Figure 1g. For the detection of thrombin, a dye-labeled human thrombin aptamer was employed in the GO platform. The dye-labeled aptamer/GO complex shows a strong selectivity to human thrombin and can detect human thrombin at different concentrations (Figure 1h), which illustrates the generality of GO as a platform that can detect biomolecules sensitively and selectively. 

**Figure 2:**
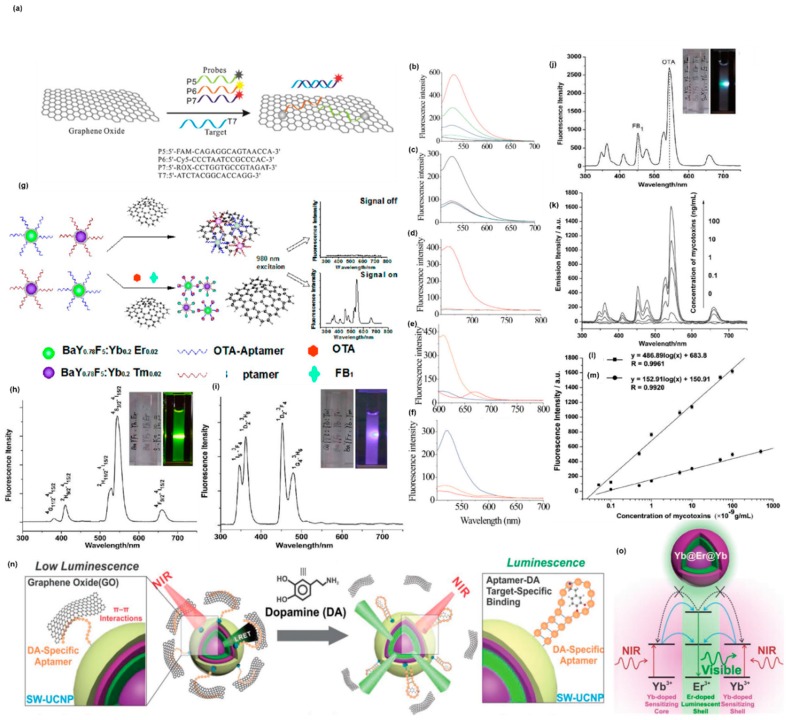
(**a**) GO-based multicolor DNA analysis. (**b**) Fluorescence intensity of P1 in response to GO (black), GO+T1 (red), GO+T2 (cyan), GO+T3 (blue), and GO+T4 (green). (**c**) Fluorescence response of P1 (10 nM) GO mixture to the fully complementary target T1 (black) and to a single-base mismatch target: M1 (red), M2 (blue), and M3 (green). (**d**–**f**) Multicolor detection that is shown by the response of the fluorescence peaking at different spectra for different targets: T5 (blue), T6 (red), and T7 (orange). (**g**) Design of the Up-conversion nanoparticles (UCNPs)-aptamer/GO sensor. The fluorescence spectra emitted by BaY_0.78_F_5_:Yb_0.2_, Er_0.02_ UCNPs (**h**), BaY_0.78_F5:Yb_0.2_, Tm_0.02_ UCNPs (**i**), and mixed UCNPs (**j**). (**k**) Multiplex detection of the UCNPs-GO aptasensor in the presence of both OTA and FB_1_. The fluorescence intensity plotted against the concentration of ochratoxin (OTA) (**l**) and FB_1_ (**m**). (**o**) The detection process of dopamine by the sandwich-structured UCNP. (**p**) Sandwich-structured UCNP and the prohibited energy-back transfer. Reprinted with permission from References [78,80,81].

**Figure 3:**
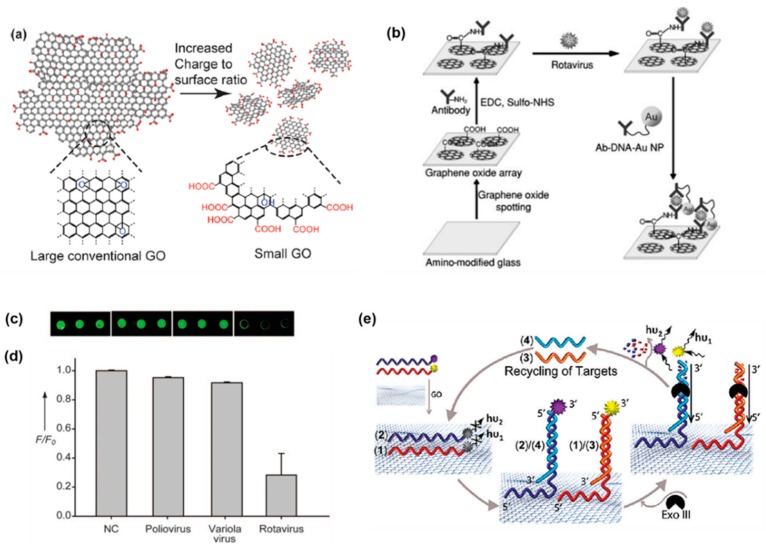
(**a**) Conventional GO and nGO used as artificial protein receptor exhibiting OCG on the edges which are binding sites for biomolecules. (**b**) Nano GO-based immuno-biosensor. (**c**) Nano GO-based immune-biosensor. Fluorescence signals of the negative control (NC), poliovirus, and variola virus were similar. The fluorescence signal of the rotavirus was significantly reduced. (**d**) Quantitative data showing that the fluorescence quenching effect caused by the target rotavirus was 15 times higher. (**e**) Exo III-induced recycling analytes that amplify sensing signals and the multiplexed analysis on GO platform. Reprinted with permission from References [75,79,83].

The effect of a longer unpaired region in the target ssDNA with GO was evaluated by He et al. by comparing the fully complementary target DNA (17 bp) with three different target DNAs (47 bp) [78]. P1 is a FAM-labeled oligonucleotide/GO complex (FAM was tagged at the 5′end) that can work as an ssDNA probe. T1 is a fully complementary targeting ssDNA of P1 that can form a duplex with P1. T2, T3, and T4 are longer target DNAs of which the complementary regions for P1 were designed in the middle, at the 5′ end, and the 3′ end, respectively. While the fluorescence intensity of T4 was significantly higher compared with T2 and T3, all the longer targets were observed to produce weaker fluorescence signals compared T1 (Figure 2b). The 5′ end tagged FAM was located within the duplex region after being hybridized with T4, which made it more likely to detach from the GO surface. Whereas, it would be located at the junction of the ssDNA and the dsDNA region when being hybridized with T2 and T3, which caused partial adsorption onto the GO surface due to the ssDNA region. The sensitivity of this DNA probe to a single mismatch was also studied and it was found that a single mismatch resulted in a reduction in the fluorescence signal (Figure 2c), which allowed a single-nucleotide polymorphism (SNPs) analysis. In addition, the fact that a single mismatch resulted in a reduction in fluorescence of the sensor is more significant than that of the GO-based sensing platform reported by Lu et al., indicating that the sensor possessed a higher sensitivity to single-base mismatches. Three probes (P5, P6, and P7) were labeled with FAM, which emits a blue color, orange colored cyanine 5 (Cy5), and red colored ROX at the 5′ end, respectively. The three probes bind to three different types of tumor-suppressor genes (p16, P21, p53, respectively). A mixture of the three probes was added to the GO solution. Binding of the target to one probe resulted in the labeled fluorophore emitting its own colored fluorescence. However, fluorophores with the other two colors barely emitted (Figure 2c–e), which achieved multicolor fluorescence DNA analysis based on aptamer/GO design and provided a prospective approach for multiplex detection. However, the three fluorophores were excited at different wavelengths and the emission peaks were too close to each other to be distinguished, which would not facilitate simultaneous detection of multiple targets. The exonuclease III (Exo III) is an enzyme which selectively digests a duplex DNA from the 3′ end but leaves the complementary strand intact. Taking the advantage of Exo III and a label at the 3′ end of a long probe that can form an ssDNA/duplex structure with the 3′ end complementary region, the amplified detection of DNA through recycling targets was achieved by Willner’s group (Figure 3e). The detection limit for ssDNA was increased to $5\times 10^{-12}$ M [79]. The authors also elucidated a multiplex analysis of the DNA sensor through using a unique combination of different probes and different fluorophore labels using the concept of “OR” and “AND” logic gates.

Being easily adsorbed by biological samples, inducing autofluorescence and light scattering are the drawbacks for conventional organic fluorophores. Up-conversion nanoparticles (UCNPs) can overcome the drawbacks of traditional organic fluorophores. Accordingly, they possess the advantages of a large signal-to-background ratio and can resist photofading and blinking. Wu et al. used the UCNPs as novel fluorescent labels in a GO-based aptasensor to simultaneously detect different types of mycotoxins (Figure 2g) [80]. The UCNPs were first modified by amino groups and then conjugated with avidin. Thus, the UCNPs could be linked to biotin modified aptamers through the biotin-avidin affinity reaction. Using different doping agents (Yb and Tm, respectively), two types of UCNPs (BaY_0.78_F_5_: Yb_0.2_, Er_0.02_ and BaY_0.78_F5:Yb_0.2_, Tm_0.02_), were excited at a wavelength of 980 nm but emitted different fluorescent signals visible to the naked eye as different colors (green and purple, respectively) (Figure 2h,i). Besides, the emission peak of one UCNP did not overlap with that of the other one in the fluorescence spectra of the mixed UCNPs, and the mixture of the two nanoparticles gave off a mixed color of the two (Figure 2j). This met the requirement of a multiplexed sensing system. Two varieties of mycotoxins: ochratoxin (OTA) and fumonisin B_1_ (FB_1_) served as targets to test whether UCNPs enable the GO-based aptasensor to detect different types of targets simultaneously. Aptamers of OTA and FB_1_ were labeled by BaYF_5_:Yb Er and BaYF_5_:Yb Tm UCNPs, respectively, indicating the ability of multiplexed sensing for various targets. In the fluorescence spectra of the UCNPs-GO sensor, the simultaneous addition of OTA and FB_1_ induced fluorescence emission with two discrete peaks at 542 nm and 452 nm that corresponded to OTA and FB_1_, respectively (Figure 2k), which proved the multiplex detection of the UCNPs-GO aptasensor. In addition, the fluorescence intensity of the UCNPs-GO sensor exhibited a linear relationship with the concentration of the two mycotoxins within the concentration range of 0.05 ng·mL^−1^ to 0.1 μg·mL^−1^ and 0.1 ng·mL^−1^ to 0.5 μg·mL^−1^ for OTA and FB_1_, respectively. According to the statistical analysis, the UCNPs-GO sensor showed a different detection limit for OTA and FB_1_, which was 0.02 ng·mL^−1^ and 0.1 ng·mL^−1^, respectively. However, the up-conversion efficiency of conventional lanthanide-doped NPs was low due to the energy back-transfer form excited Er ^3+^ to Yb ^3+^, requiring laser excitation with high power density for compensation at the expense of biocompatibility. Rabie et al. developed a UCNP consisting of a luminescent activator shell doped with 2% Er sandwiched between an inner sensitizing core doped with 20% Yb and an outer sensitizing shell doped with 20% Yb (Figure 2o) [81]. The luminescent shell was designed to be 10 nm so that the distance between Er that was doped at the innermost side of the luminescent shell and Yb doped at the core was less than 5 nm, guaranteeing the effective energy transfer from Yb to Er. The thickness of the outmost sensitizing Yb shell that covers the hole NP was also 10 nm, which was proven to be able to enhance the FRET efficiency [82]. A silica coating was used to facility the surface chemistry modification and to protect the sandwich structured UCNP from quenching by water. A dopamine aptamer was then anchored to the UCNP, enabling detection of dopamine using GO as a quencher. It was found that the sandwich-structured UCNP could successfully prevent back-energy transfer due to spatial separation of Yb and Er, and the efficiency of FRET was high enough to detect dopamine with a concentration down to pM. 

GFM usually form aggregates easily under physiolological conditions due to the bad polydispersity, which impairs the interaction with biomolecules and the sensing efficiency. This issue was overcome by Chou et al. using nanoscale GO (nGO) as artificial receptors [75]. The diameters of nGOs were uniform and enhanced the zeta potential. The nGO had an increased edge to basal plane ratio resulting in an increased OCG density. Thus, nGOs are less likely to aggregate as molecular adsorption on GO and other carbon material presents an edge to basal plane preference. Thus, an increase in edge to basal plane ratio can enhance the biomolecular response. Besides, the increased OCG also resulted in a higher charge density, which favored electrostatic interaction with the biomolecules (Figure 3a). Furthermore, the small surface area made highly flexible nGO planar scaffolds that can interact better with biological macromolecules with complex 3D structures. As was mentioned before, nano-GO showed photoluminescence within the visible to NIR range. Taking advantage of this unique property, a GO-based label-free immune-biosensor for pathogen detection was synthesized by Seo’s group (Figure 3b) [83]. A rotavirus was used as a pathogen model in this study. Negatively charged GO sheets were bound to the amino-modified surface, which was positive through electrostatic interaction. The antibodies of the rotavirus can bind to the carboxyl group of GO via carbodiimide-intermediate coupling, facilitating capture of the rotavirus by the GO/antibody through antigen–antibody interaction. Gold nanoparticles (AuNP) have been used as a quencher for oxidized carbon nanotube GO photoluminescence due to FRET between the GO sheets and AuNPs [84], which implies that AuNP is a potential quencher for GO. To realize the highly sensitive and selective GO-based immune-biosensor, an AuNP-DNA-antibody complex was thus synthesized. AuNPs and the antibody were linked through 100-mer ssDNA molecules which modulate the distance between the antibody and the AuNP. Thus, the AuNP could be placed close to the GO surface. A significant reduction in fluorescence of GO due to quenching caused by the selective bounding of AuNP-DNA-antibody to target cell was achieved, enabling efficient identification of pathogenic cells (Figure 3c,d). 

## 4. GFM-based Field Effect Transistors (FET) 

**Figure 4:**
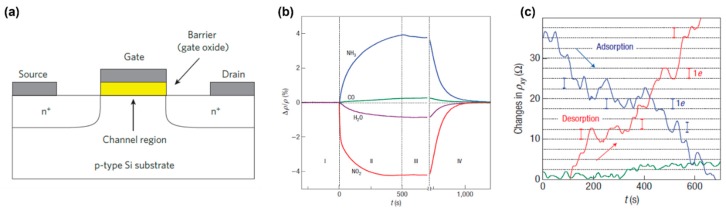
(**a**) The cross-section of a FET. (**b**) Relative change in resistivity of single layer graphene (SLG) at zero magnetic fields exposed to different diluted gas molecules with a concentration of 1ppm. The positive signals indicate electron doping while the negative ones indicate hole doping. Region I: Resistivity change of SLG in a vacuum; II: SLG exposed to diluted gas molecules; III: Expulsion of gas molecules; IV: SLG annealed. (**c**) Change in the Hall resistivity ($\rho_{xy}$) of SLG due to adsorption (blue curve) and desorption (red curve) of a single NO_2_ molecule (adsorption). The green curve shows the response of SLG to pure He. Preprinted with permission from Reference [16,89].

FET is a device concept in electronics widely applied in sensors due to the incomparable success. FET is usually composed of a source and a drain electrode with a channel region connecting them, and an insulating barrier that forms a separation between the gate and the channel (Figure 4a) [16]. Operation of FET relies on changes in channel conductivity reflected by the drain current ($I_{D}$) and controlled by an electric field caused by the voltage applied between the gate (top or bottom) and the source (V_GS_). FETs with a high quality are required to react fast to changes in V_GS_, which can only be satisfied by short gates and quick movement of carriers in the channel. However, short gates always bring about adverse impacts such as degraded electrostatics and short-channel effects. According to the prediction made by the scaling theory, those impacts can be suppressed by a FET that has a thin barrier and is controlled by a thin gate. Thus, graphene is an appealing material for use as a transistor as graphene makes it possible to fabricate channels that are just one atom thick. Besides, graphene shows ballistic conduction of carriers without scattering, typical linear I-V characteristics, and sustainability to extremely high current densities ($>10^{8}$ A/${\mathrm{cm}}^{2}$) [1]. The on–off resistance ratio of a graphene transistor is modest (less than 30 at 300 k) but is adequate for a logic circuit. The moderate rate is due to the limited carrier excitation under limited temperature and is a limitation shared by all materials without a bandgap exceeding $k_{B}T$. The emerging need for transparent and flexible electronics challenges the traditional manufacturing processes. However, the development of GFMs that allow inkjet printing has overcome the challenge, as inkjet printing is a facile, low-cost, and large-scale fabrication technique. [85].

### 4.1. Gas Sensor

Several unique properties make graphene an attractive material for gas sensing. A single-layer graphene sheet is one atom in thickness, which defines it as a perfect 2D material. Thus, the whole volume of it is able to be exposed outwards to adsorb gas molecules, which improves the sensing efficiency [86]. Graphene has a superior conductivity, that is comparable to metal, and a low intrinsic electrical noise due to the unique 2D crystal structure with few crystals defects and a large conjugated π system. Therefore, extra electrons in small quantities can lead to a significant change in graphene’s conductivity [1,87,88]. Besides, even single gas molecule adsorption can be detected by a graphene sheet and it causes a step-like change in the resistance of graphene (Figure 4c) [89]. Furthermore, the detecting signal of a single-layer graphene sheet (SLG) will never be saturated if the SLG is exposed to gas molecules at a low concentration because chemical doping affects graphene in a cumulative manner. 

Gas molecules interact with graphene in different modes and thus gas molecules affect the properties of SLG in different ways. Schedin used the Hall effect technique [89] to study the interactions. The resistivity of SLG (ρ=1/σ) which is equal to the zero-field longitudinal resistivity ($\rho_{xx}$) increases when ammonia and CO are adsorbed but decreases when water and nitrogen dioxide are adsorbed (Figure 4b). According to their Hall measurements, nitrogen dioxide, water vapor, and iodine work as acceptors. However, ammonia, carbon monoxide and ethanol behave like donors. The same result was reported by Leenearts et al. By performing first-principles calculations using density functional theory, they proved that NO served as a donor [90]. The sensing regime of SLG usually consists of a rapid response region at the beginning and the post-saturation region where the detection signal changes slowly [89]. Though SLG has a large contacting surface area, it still has a finite size. Thus, only a limited amount of gas molecules can interact with the micron-sized sensitive area. Expulsion of gas molecules cannot result in rapid and complete recovery in resistivity. Annealing at 150 °C in a vacuum or a short exposure to ultraviolet can achieve complete recovery.

**Figure 5:**
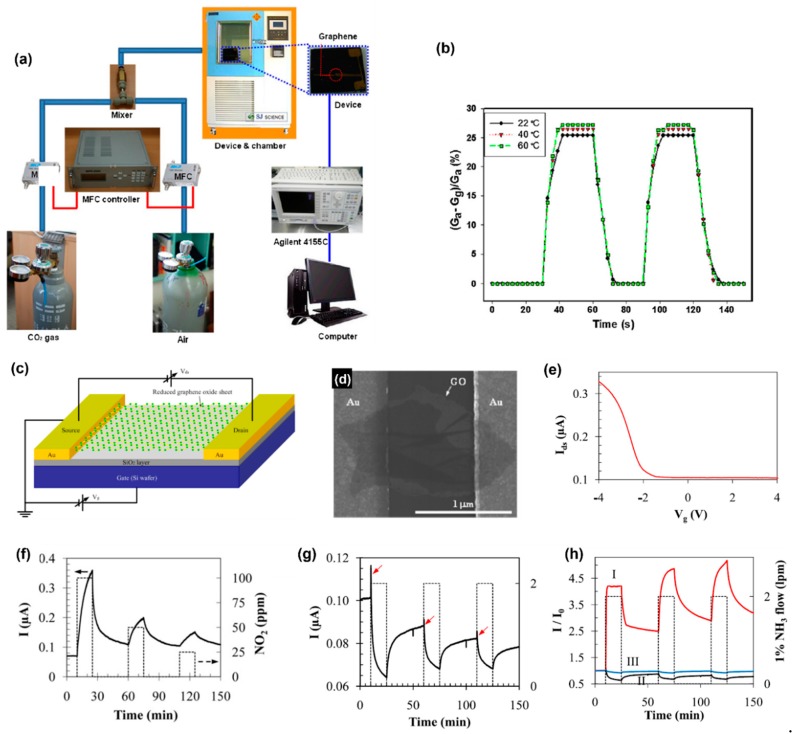
(**a**) Experimental setup for CO_2_ gas sensor. (**b**) Real-time response of the sensor in conductance at 100 ppm CO_2_ gas at different temperatures. (**c**) rGO gas sensor. (**d**) Scanning Electron Microscope (SEM) image showing a deposited GO sheet that bridges the gap between two Au electrodes. (**f**) The I_ds_-V_g_ curve of the multi-step annealing fabricated sensor. (**e**) Detection of NO_2_ at different concentrations by a multi-step heating fabricated rGO sensor at room temperature. (**f**) Detection of 1% NH_3_ by a one-step annealing fabricated rGO sensor. (**g**) I: Response of rGO sensor for 1% NH_3_ obtained from (**h**), II: Response of the reused rGO sensor after 2 months for 1% NH_3_ III: Response of the reused rGO sensor treated with another heating. Reprinted with permission from References [91,92].

Yoon et al. reported a high-performance graphene sheet-based FET carbon dioxide (CO_2_) gas sensor [91]. The graphene sheet was exfoliated through a stamping method by using a sticky cured poly-dimethylsiloxane (PDMS) as the stamp. Graphene flakes were then stamped on to a Si substrate with a thermally oxidized surface that had Au alignments on top. Drain and source gold electrodes were fabricated through electron beam lithography (EBL) using polymethylmethacrylate (PMMA) as the electron beam resistance material, deposition of a gold layer through electron-beam evaporation, and afterward liftoff via a remover PG solution. To test the detection of CO_2_, the device was placed into a chamber that was maintained at a constant temperature and constant humidity and controlled the concentration of CO_2_ by adjusting the flow rate of CO_2_ and compressed air; the relative changes in conductivity were recorded (Figure 5a). The relative conductance change increased upon the adsorption of CO_2_ gas molecules (Figure 5b), which indicates charge transfer between CO_2_ and the graphene sheet. Furthermore, the relative change in conductance was linearly related to the concentration of CO_2_. The physically adsorbed CO_2_ molecule served as an acceptor on the graphene sheet. The authors stated that the interaction between the CO_2_ molecule and the graphene sheet was different from other molecules like NH_3_ and NO_2_ since graphene exhibited a fast response (8 s) and recovery (10 s) for a CO_2_ molecule. Besides, the desorption of CO_2_ gas molecules can be achieved without applying high-temperature annealing. A similar FET sensor was fabricated by Lu et al. using rGO (Figure 5c), and three terminal FET measurements were conducted to demonstrate the p-type semiconductor behavior of rGO beside the two-terminal DC measurements (Figure 5e) [92]. GO sheets were deposited on wafers with interdigitated electrodes through casting drops of a GO suspension onto the electrode after water evaporation (Figure 5d). Two thermal reduction strategies of GO were applied: successive multi-step heating, including three cycles for 1 h at different temperatures (100 °C, 200 °C and 300 °C), and one-step heating at 200 °C under an Ar flow for 2 h. The carrier transport behavior of rGO was characterized and it was found that the increase in V_g_ led to a decrease in I_ds_, which suggested rGO was dominated by holes. The p-type semiconductor behavior was possibly due to defects and adsorption of molecules in the atmosphere (such as H_2_O and O_2_) after fabrication. The result is consistent with Novoselov et al.’s study on the field effect of few-layer graphene (FLG) [1]. The peak of *ρ* was found to shift towards a large positive $V_{g}$, indicating that FLG can behave like a whole metal. Successive multi-step heating resulted in an rGO with a higher conductivity and sensitivity to NO_2_. The response (15 min) and recovery (25 min) time of the sensor to NO_2_ were longer than that of Yoon et al.’s sensor, and the clean air flow was sufficient for the recovery. The conductance of the sensor increased upon the adsorption of NO_2_ and the sensing signal was dependent on the NO_2_ concentration (Figure 5f). RGO exhibited p-type behavior, and NO_2_ has a strong electron-withdrawing power as a strong oxidizer; once NO is adsorbed on to rGO, there would be a transfer of electrons from rGO to NO_2_, which would result in an increase in the hole concentration and the electrical conductance of rGO. In contrast, adsorption of NH_3_ decreases rGO conductance since NH_3_ acts as electron donor that decreases the concentration of holes in rGO. The sensing signals for NH_3_ and NO_2_ of this rGO sensor were consistent with Schedin et al.’s research. The sensor fabricated via a successive multi-step reduction showed an extraordinary long recovery time (50 h) for NH_3_, which agreed with other rGO NH_3_ sensors. The sensor fabricated via one-step heating had a better performance. However, the sensor (200 °C) showed several abnormal NH_3_ sensing behaviors. An abrupt current increase can be observed in each sensing cycle, which could be attributed to an unstable gas flow in the chamber caused by gas alternating and other incidental noises (Figure 5g). Besides, if the sensor was reused after 2 months, the sensing signal of it showed the complete opposite trend (Figure 5h). The sensing signal was restored to the original trend but at a weakened strength after the sensor was reheated at 200 °C in Ar (Figure 5h). The abnormal behavior may be caused by a non-ohmic contact between the GO and Au electrodes that is suggested by the asymmetric and nonlinear I_ds_-V_ds_ curve. Li et al. developed a graphene-based FET sensor with a graphene decorated electrode and a palladium-modified rGO (Pd-rGO) that was deposited on a Si/SiO_2_ wafer for NO detection [93]. Patterned nickel electrodes were fabricated on a wafer via photolithography and the lift-off technique and were then coated with graphene through the chemical vapor deposition (CVD) growth of graphene method. To fabricate Pd-rGO, an rGO solution was first prepared through the reduction of GO by hydrazine and was then added into a solution that contained Pd nanoparticles. According to the authors, graphene-coated electrodes and Pd modified rGO resulted in an enhanced contact. Thus, the issue that GFM based FET sensors usually have a high resistance due to the GFM-on-electrode configuration caused poor electrical conducts between the metal electrode and GFM can be solved. Besides, the sensitivity of the sensor was improved as the graphene contact increased the carrier transfer mobility. In addition, Pd nanoparticles can serve as an absorption medium of NO causing symmetric Schottky barriers at the interface between the Pd nanoparticles and rGO. A Pd-rGO solution was dropped onto the wafer and an alternating current (ac) voltage was applied to the electrode to deposit Pd-rGO between the electrodes. The ac dielectrophoresis (ac-DEP) provides a strong electric field gradient so that Pd-rGO sheets can be aligned along the field direction between the source and drain electrodes. It was found that the Pd-rGO sheets of the device treated with ac-DEP weredeposited between the two electrodes in an orderly manner forming conductive channels, whereas, Pd-rGO sheets deposited by simple drop casting distributed arbitrarily all over the wafer surface leading to insulation. The conductance of the device increased with the adsorption of NO, indicating that NO acts as an acceptor, which agrees with Chen et al.’s study. [94] A mild current annealing was applied to the sensor to decrease the recovery time (~1000 s) at the expense of sensitivity. Current annealing removed some oxygen-containing groups, which are responsible for the irreversible sensitivity to NO gas. Carrier scattering, which impedes the mobility of carriers can be also reduced by current annealing, and therefore conductance of graphene was significantly improved [95]. The response time of the sensor was 240 s. Dan et al. fabricated a graphene FET vapor sensor in the same way as Yoon et al.’s study except that they used a ’clean’ process, which was achieved by placing the device in a reducing atmosphere (H2/Ar) at 400 °C for 1 h to remove the 1 nm thick contamination layer, which was residue of resist material on the graphene produced during the EBL process [96]. They concluded that the clean process could effectively restore the intact structure of graphene and enhance the electronic properties of graphene. However, the clean process resulted in a sharp decrease in sensor response because the contamination layer served as an absorptive layer that kept gas molecules close to the surface of the p-type graphene transistor. Besides, the chemical doping by water and oxygen in the environment, which may be the cause for p-type behavior of graphene, was also reduced by the clean process. The sensitivity of the sensor to various kinds of vapor was tested. Water vapor is an oxidant; besides, it leads to p-type behavior. Thus, adsorption of additional water vapor increases conductivity. Octanoic acid increased the hole concentration the conductance of graphene. According to the authors, it would deprotonate and would act as an electron acceptor when adsorbed on water doped graphene. Whereas, trimethylamine is an acceptor for protons while coexisting with the adsorbed water. Therefore, the conductance of graphene decreased.

### 4.2. FET Biosensor

**Figure 6:**
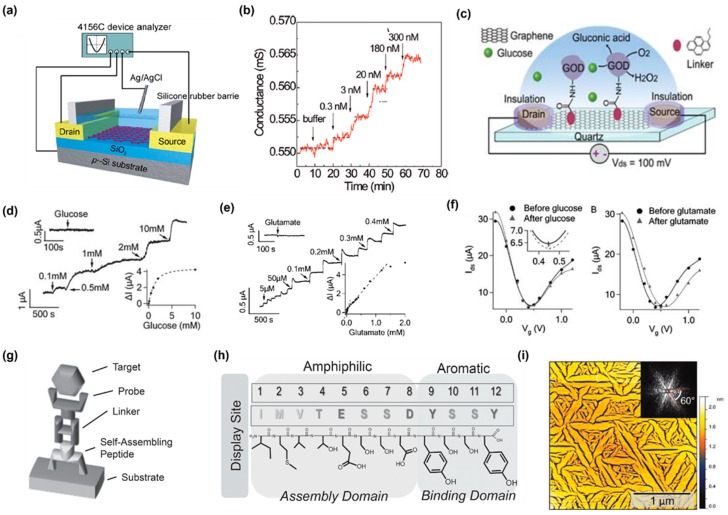
(**a**) Electrolyte-gated graphene FET for protein. (**b**) Real-time conductance changes of the sensor upon exposure to BSA with different concentration. (**c**) Schematic illustration of glucose oxidase (GOD) modified graphene. (**d**) The response of the glucose sensor $I_{D}$ to glucose at various concentrations. The upper insert presents that graphene FET without GOD functionalization shows no response to glucose. The lower insert fits the response of the device and the concentration of glucose into an exponential function. (**e**) The response of the glutamic dehydrogenase (GluD) modified sensor to glutamate of different concentrations in the presence of beta-Nicotinamide adenine dinucleotide (*β*-NAD). (**f**) Right: transfer curves of GOD modified graphene with and without the addition of glucose (10mM). Left: transfer cures of GluD modified graphene with and without the addition of glutamate (1mM) in the presence of *β*-NAD. (**g**) Design of the GrBP5-WT peptide functionalized substrate. (**h**) Chemical structure of GrBP5-WT sequence (**i**) Atomic force microscope (AFM) image of the self-assembled highly ordered peptide on graphite. Insert: FFT of the AFM image. Reprinted with permission from References [99,100,103,104,105].

As mentioned above, various modifications are applicable to GFM, which makes GFMs a good platform for biomolecules. The binding of biomolecules to probes immobilized on a graphene surface will alter the electric charge distribution and the charge carrier density on the graphene surface, leading to change in conductance of the channel and a Dirac point shift. Besides, GFMs have an affinity for some biomolecules and binding also can cause a change in conductance. Thus, graphene is an ideal material for FET-based biosensors. Compared with fluorescence aptamer/graphene sensors, which emit a fluorescence signal, the electrical signal operated FET biosensors are more accurate and reliable. Besides, most FET-based biosensors are label-free, which overcomes the drawbacks caused by the labeling step such as low labeling efficiency, complicated multi-step analysis, and sample contamination [97,98]. Ohno et al. developed an electrolyte-gated graphene FET that can detect a protein (Figure 6a) [99,100]. Graphene was mechanically exfoliated from graphite and transferred to a SiO_2_/Si substrate of which the SiO_2_ layer was fabricated through thermal growth by scotch tape. The source and drain electrodes were assembled in the same way as Yoon et al.’s device except that Ti/Au was the metal used for the electrodes. The silicon substrate was degenerately doped and acted as a back gate. The device was immersed in an electrolyte. To make sure the surface of the graphene channel was able to be filled with electrolytes and analytes, a silicon rubber barrier was placed around the device as a container. An Ag/AgCl reference electrode was inserted into the electrolyte working as a top-gate electrode that can remove major sensing artifacts through manipulation of solution potential. The conductance of the device immersed in an electrolyte and the device in a vacuum were studied. The transconductance of the device immersed in the electrolyte was 36 times higher than that of the device in the vacuum. The top- and back-gate capacitance of the immersed device were then estimated by the authors using a simple model that the electrolyte-gate graphene FET capacitance should be considered as a series of electrostatic gate capacitances and quantum capacitances. The top-gate capacitance was found to be three orders of magnitude larger than the back-gate capacitance. The top-gated graphene FET formed an electrical double layer at the graphene–solution interface of which the thickness was determined by ion size (1–5 nm) according to the Debye–Hu¨ckel equation, whereas, the oxide layer of silicon, which is the insulator between double layer for the back-gate graphene FET was several orders of magnitude thicker (~300 nm) [99,101]. Besides, the dielectric constant of the solution ($\varepsilon_{r}$) was much higher than SiO_2_ ($\varepsilon_{r}\;$= 3.9) [99]. The authors concluded that the superior charge carrier transferring characteristic of electrolyte-gated graphene FET derived from the high top-gated capacitance. Since top-gated (solution-gated) graphene FET has the advantage of a higher transconductance and better field-effect characteristics, most GFM FET biosensors and chemical sensors are top-gated. The device was immersed in a phosphate buffer with a pH of 6.8 to examine the direct aptamer-free detection of a bovine serum albumin (BSA) biomolecule, which is negatively charged in the buffer since the isoelectric point of it is 5.3. The adsorption of negatively charged BSA to graphene increased the hole carrier density, and thus the conductance of the device (Figure 6b). However, the change in conductance was relatively small, which was owing to the two-terminal measurement, desorption of BSA molecules, and uncharged amino acids in BSA due to a minor difference in pH between the buffer and the isoelectric point. The authors also developed an aptamer modified graphene FET immunosensor for the detection of immunoglobulin E (IgE) [102]. The sensor was fabricated in the same way as a previously reported device. The graphene surface channel was cleaned through annealing in Ar/H_2_ at 300 °C for the preparation of the immobilized IgE aptamer. 1-Pyrenebutanoic acid succinimidyl ester (PASE), a linker widely applied in CNT functionalization, was used to achieve the noncovalent functionalization of the graphene surface without introducing defects to the graphene. The pyrenyl group of the linker can conjugate to the graphene basal plane through stacking and can link to the amine group of the IgE aptamer via carbodiimide-mediated coupling. The height of the aptamer was smaller than 3 nm so that protein-aptamer reactions can occur within the electrical double layer of which the thickness was the Debye length. If charged molecules are located more than a Debye length away, they cannot affect mobile charges in a transistor’s channel any more. Thus, the sensor can successfully detect the binding of IgE to its aptamer. The binding of positively charged IgE to the negatively charged aptamer will cause a decrease in conductance. The adsorption of sensing molecules onto both devices developed by Ohno et al. follows the Langmuir adsorption isotherm. Glucose oxidase (GOD) can also be immobilized to a graphene sheet through the PASE linker. In this way, Huang et al. developed a GOD modified graphene-based FET for real-time glucose detection (Figure 6c) [103]. Graphene was grown via CVD on a Ni film that was evaporated on a SiO_2_/Si substrate and transferred to a quartz substrate with the aid of (poly(methyl methacrylate) PMMA. PMMA was then removed by chlorobenzene and acetone. Graphene was also cleaned through annealing before being functionalized by GOD. To immobilize GOD, a GOD solution was incubated with a linker-modified device in a Na_2_CO_3_–NaHCO_3_ buffer (pH = 9) overnight at 4 °C. GOD catalyzes the oxidation of glucose (*β*-D-glucose + O_2_ + H_2_O → D-glucono-1,5-lactone + H_2_O_2_) and the oxidation reaction of glucose results in an increased conductance of the device (Figure 6d). The response of the device to glucose is saturated when the concentration of glucose is over 10 mM because there are limitations in the density of the immobilized GOD and in the GOD reaction rate. Glutamate, a major neurotransmitter released by neurons, can be oxidized by glutamic dehydrogenase (GluD) in the presence of *β*-NAD (L-glutamate + *β*-NAD + H_2_O ↔ α-ketoglutarate + *β*-NADH + NH_4_^+^). Taking advantage of a similar principle, a glutamate sensor was fabricated using GluD immobilized graphene by the authors. The reaction of the GluD immobilized graphene FET sensor with glutamate in the presence of *β*-NAD also caused an increase in conductance of the sensor (Figure 6e). The transfer of the electrons produced by both the oxidative reaction of graphene should have caused a decrease in the conductance of graphene and a negative shift of the Dirac point (minimum conductance) of the transfer curve. However, the addition of both glucose and glutamate resulted in a shift to the right of the transfer curve, suggesting a P-type doping effect of both molecules (Figure 6f). The glucose sensor showed a tiny shift to the right of the transfer curve compared to the GluD sensor, consistent with the poorer performance of the glucose sensor. The authors stated that the increase of graphene conductance was probably due to the products created from the oxidative reactions. They proved that the graphene conductance can be increased by H_2_O_2_ and NH_4_OH, which are products of glucose and glutamate oxidation respectively, whereas, graphene showed no response to D-glucono-1,5-lactone and α-ketoglutarate, which are also products of the two oxidative reactions respectively. They also fabricated SWCNT-based FET sensors for the detection of glucose and glutamate in similar manners; however, both SWCNT-based sensors showed a weaker signal. 

The amino acid sequence of the graphene binding dodecapeptide (GrBP5-WT) is IMVTESSDYSSY. The GrBP5-WT is unique because it undergoes self-assembly to form a long-range-ordered monolayer structure on the atomically flat solid surface of the graphitic material [104]. The peptide is composed of two distinct domains: the aromatic domain, which is responsible for the strong π–π stacking interaction with graphene and is crucial for cluster formation during the initial aggregation stage; the amphiphilic domain, which plays the main role in self-assembly of the peptide (Figure 6h). The self-assembled highly ordered peptide structure was observed to display a six-fold symmetry (Figure 6i) [105], which is probably because the molecular recognition with the underlying hexagonal graphitic lattice guides the self-assembly of the peptide along the specific crystallographic directions. The interaction with the substrate and self-assembly can be tuned via sequence mutations. The surface chemistry of BrBP5-WT can be modified through the addition of specific amino acids. A 4-probe graphene FET was functionalized by the co-assembled biotinylated BrBP5-WT mutant, which was modified by biotin (Bio-GrBP5) and the SS-GrBP5 mutant, which presents hydrophilic chemistry [105]. The 4-probe design can eliminate the effect of contact resistance at the interface with electrical leads during measurement. The biotinylated mutant was used for the detection of streptavidin (SA). To achieve the clinical potential of the sensor to test biomarkers, serum albumin, as a major blood component, is the background protein of which non-specific adsorption needs to be prohibited. The hydrophilicity of the self-assembled SS-GrBP5 mutant surface with a contact angle of 36° has been proven by the author’s previous study [104]. The contact angle is similar to many other anti-fouling systems [106], indicating that the SS-GrBP5 assembled monolayer should also show anti-fouling properties. It was shown that signals due to non-specific adsorption of BSA by the SS-GrBP5 monolayer modified graphene FET were successfully impaired. In addition, the bio-GrBP5 and SS-GrBP5 co-assembled surface was shown to be able to detect SA against a BSA background. The resistance of the device reduced with the adsorption of SA, and it was explained by the authors that ionic and partial changes of the self-assembled GrBP5 peptide molecules, due to the interaction with the target peptide, increased the doping of graphene. 

**Figure 7:**
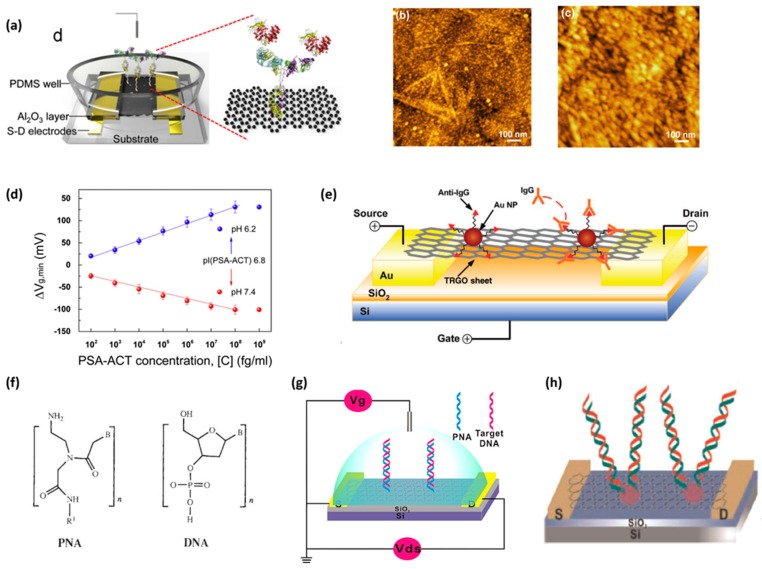
(**a**) prostate specific antigen/a1-antichymotrypsin (PSA-ACT) complex biomarker sensitive rGO FET. (**b**) AFM image of rGO surface after being treated with the PASE linker. (**c**) AFM image of the rGO surface after PSA mAb immobilization and after ETA treatment. (**d**) the electrical conductivity response of the rGO FET to the PSA–ACT complex at different concentrations. Two sets of PSA-ACT complex solutions were tested (pH = 6.8 and pH = 7.4). (**e**) AuNP anti-IgG conjugates immobilized rGO FET for the detection of IgG. (**f**) The chemical structure of DNA and PNA. (**g**) The RGO FET sensor with the PNA probe for DNA detection. (**h**) AuNP decorated rGO FET sensor for the detection of microRNA. Reprinted with permission from References [107,110,111,112,113].

Kim et al. developed a label-free rGO FET for the detection of prostate specific antigen/a1-antichymotrypsin (PSA-ACT) complex biomarker down to the femtomolar level (Figure 7a) [107]. A glass substrate with a hydroxylated surface was firstly prepared via treatment with oxygen plasma. Then, the substrate was treated with APTMS to create an amine group modified surface; in that way, GO can bind to the substrate through electrostatic interactions. After that, GO was reduced by hydrazine vapor and was annealed. The Ti/Au source/drain electrodes were fabricated on the selected area through thermal evaporation with the aid of a metal shadow mask. The Ti/Au source/drain electrodes were capped with an Al_2_O_3_ layer via thermal evaporation to protect the electrodes from the direct interaction with the biomolecules and were then encapsulated by a PDMS layer. The Al_2_O_3_ layer and PDMS well can minimize the electrode-electrolyte leakage current between the source and gate electrodes to an undetectable level. The whole device was surrounded by a PDMS well. A PASE linker was used to immobilize the monoclonal antibody prostate-specific antigen (PSA mAb) to the rGO surface (Figure 7b,c). The device was treated with ethanolamine (ETA) to avoid non-specific binding. A Pt reference electrode was introduced to the device. The isoelectric point of the PSA-ACT complex was 6.8. The authors performed the measurement in a PBS solution at different pH levels (pH = 7.4 and pH = 6.2). The conductivity of the device decreased with the binding of the PSA-ACT complex in the pH = 6.2 analyte solution where the complex was positively charged. However, binding of the negatively charged complex due to the pH = 7.4 analyte solution resulted in an increase in the conductivity of the device (Figure 7d). Charge carrier transfer that causes a doping effect will lead to the opposite result. Thus, it cannot be used to explain the conductivity change of rGO here. The authors suggested that the charged impurity scattering model might be the underlying mechanism, as rGO nanosheets have high-density defects and impurities. It has been reported that Ag NPs could non-covalently bind to graphene due to strong van der Waal interactions [108], suggesting the potential of Au NPs of which the size is similar for Ag NPs to bind with graphene. Anti-Immunoglobulin G (anti-IgG) labeled Au NPs (Au NP-antibody) were successfully immobilized to a thermally reduced GO surface (TRGO) by simply dropping an Au NP-antibody colloidal solution on TRGO and one-hour incubation [109,110]. The functionalization method was adopted by Mao et al. to their device that was built based on the design of Lu et al.’s gas sensor (Figure 7e). The device dealt with a blocking buffer (BB) that contained 0.1% Tween 20, 0.1% fish gelatin, and 1% BSA to prevent IgG from nonspecifically binding to TRGO and the electrodes for 2 h at room temperature and then the device was washed with PBS buffer. The detection by the device was carried out under an ambient environment instead of being electrolyte-gated. After the IgG samples were loaded, one hour was given for protein binding, then the device was subject to washing and drying. Binding of IgG to the device caused an increase in the resistance. The authors also found that the performance of the sensor could be determined by a TRGO based resistance and the Au NP antibody areal density. Through tuning the two factors, a detection limit down to 0.2 ng/mL was achieved. The device maintained good performance after several washing and drying cycles. Peptide nucleic acid (PNA), a DNA analogue where the phosphorylated deoxyribose backbone of DNA is replaced by N-(2-aminoethyl)glycine, obeys Watson-Crick base-pair match with DNA (Figure 7f) [111]. Cai et al. employed PNA as DNA aptamer in their rGO FET biosensor since it had the advantage of neutrality that impaired electrostatic repulsion between dsDNA, a high affinity, and a high stability (Figure 7g) [112]. An RGO suspension was firstly prepared by hydrazine reduction then was drop cast onto a Si/SiO_2_ substrate. PNA was immobilized through the PASE linker. ETA treatment was applied to increase the specificity of the device. Hybridization by the targeting DNA led to a shift to the left of the transfer curve, suggesting a reduction in conductance. The device achieved a detection limit of 100 fM. The device could be reused after being immersed in a urea solution for 5 min and subsequent (Deionized water) DI rinsing. The authors also fabricated an rGO FET sensor for microRNA detection using a PNA probe where the PNA probe was anchored with the aid of AuNPs (Figure 7h) [113]. The rGO FET design was similar to a previous study. Immersing the device in HAuCl_4_, AuNPs were able to assemble onto the graphene surface. Then, AuNPs were functionalized by an amine group through treatment with cysteamine hydrochloride since the thiol group of cysteamine can form an S-Au bond with Au nanoparticles. The device was soaked in glutaraldehyde (GA), which was used as a linker that covalently binds amino group from PNA and amine groups from cysteamine modified AuNPs and was then immersed in a PNA solution. To block nonspecific binding, the device was immersed in an ETA solution. There was a shift to the right found in the transfer curve after rGO was decorated by AuNPs due to the p-doping effect caused by the AuNPs. GA treatment also resulted in a shift to the right of the transfer curve. However, PNA immobilization led to a shift to the left, which was attributed by authors to the n-doping on the device imposed by the interaction between graphene and electron-rich nucleobases. The detection signal of microRNA, which is also electron-rich, was reflected by a reduction in conductance. A detection limit down to 10 fM was achieved by the device. Both PNA based rGO FET sensors were able to detect a single base mismatch. Mohanty et al. developed GO FET with an amine group modified substrate that can be subjected to versatile functionalization [114]. The Si/SiO2 substrate with patterned gold electrodes was treated with oxygen plasma so that APTMS can be anchored to the surface. GO can then be immobilized onto the substrate through electrostatic interactions. To functionalize the device with DNA probes, the device was incubated with 5′-pentamine-terminated DNA and O-(7-azabenzotriazole-1-yl)-N, N, N, N′-tetramethyluronium hexafluorophosphate (HATU), which is an amide coupling agent. To achieve the bacterium detection functionality, the device was treated with valeric acid and HATU to create a hydrophobic background for selective detection. GO was then modified with an amine group via treated ethylenediamine and HATU, giving rise to positively charged graphene-amine (GA). Adsorption of the target DNA caused an increase in conductivity. The authors attributed the increase in hole density to the phosphate ions of the target DNA that caused negative-charge molecular gating. Gram-positive *Bacillus cereus* bacteria, which have a negatively charged cell surface because of the attachment of poly teichoic acid molecules on the cell wall, can be thus adsorbed to GA through electrostatic interactions. Single bacterial cell adsorption resulted in a 42% increase in conductivity. The adsorbed bacteria survived for up to 4 h. The single-bacterium resolution and good biocompatibility make the device an auspicious bio-diagnostic sensor. Bacterial detection also led to an increase in conductivity. According to the authors, the attachment of negatively charged bacteria, which could act as negative potential gating, would increase the hole density. The authors elucidated that the device was still sensitive after multiple layers of polyallylamine hydrochloride (PAH) and polystyrene sulfonate (PSS) were adsorbed onto the GO. Besides, the electrostatic adsorption of the positively charged PAH to the GO device resulted in a decrease in conductivity and the subsequent adsorption of negatively charged PSS to the GO-PAH device resulted in an increase in conductivity. For GA devices, PSS adsorption occurred first, leading to a conductivity increase, followed by PAH adsorption leading to a conductivity increase. Lerner’s group has shown the potential of manufacturing graphene FET-biosensors under commercial and industrial environments rather than in a research lab. In their design, graphene was prepared by CVD and transferred to a clean chip fabricated in a commercial (microelectromechanical systems) MEMS foundry. The graphene modified wafer was then cut into a die and packaged by a 44-pin ceramic quad flat J lead (CQFJ) [115]. To immobilize the bio-probes on graphene, a carboxyl group was first introduced to the graphene surface. The device was incubated with 4-carboxybenzenediazonium tetrafluoroborate to anchor the carboxyl group. Then, bio-probes were immobilized to the graphene via carbodiimide (NHS/EDC) intermediated coupling [116]. The device was incubated with PEG-amine so that a protective layer was formed to block non-specific surface chemistry. The device was finally incubated with ETA to quench the remaining activated carboxyl groups. The highest sensitivity of their sensor reached 2 pg/mL and the standard response ranged from 2 pg/mL to 1000 pg/mL. 

### 4.3. Chemical Sensor

**Figure 8:**
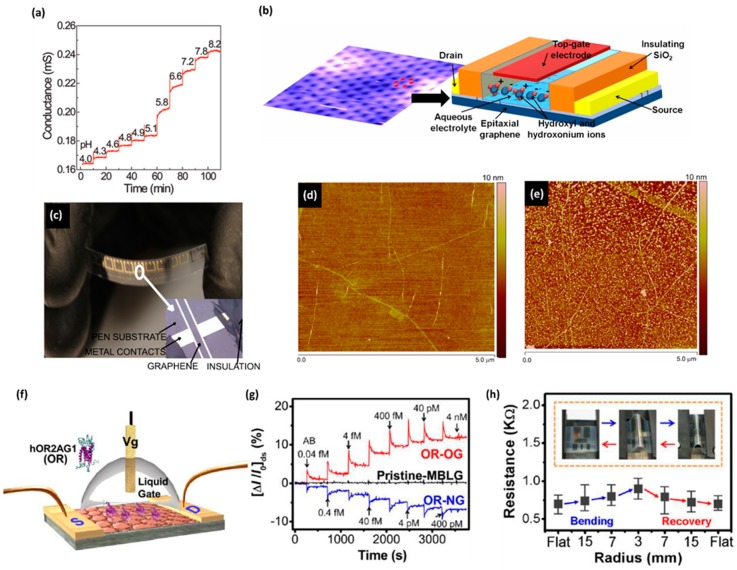
(**a**) Response of electrolyte-gated graphene FET conductance to pH. (**b**) Solution-gated epitaxial graphene FET as a pH sensor. (**c**) Photograph of solution-gated graphene FET on the PEN substrate. (**d**) AFM image of the completely cleaned graphene surface (**e**) AFM image of the graphene surface with resist residues. (**f**) Scheme of the ultrasensitive and flexible graphene FET bioelectronic nose. (**g**) The response of OG, NG and pristine bilayer graphene to the AB solution at various concentrations. (**h**) Electrical stability of the graphene bioelectronic nose. Reprinted with permission from References [99,118,119,121].

Ohno et al.’s electrolyte-gated graphene FET sensor can also work as a pH sensor [99,100]. The conductance of the sensor increases step-wisely as the pH rises from 4.0 to 8.2 (Figure 8a). It was concluded that an increase in hydroxide ions around graphene would lead to a rise in the conductance of graphene. Since hydroxide ions act as an acceptor for hole doping in CNT [117], an increased concentration of OH^-^ leads to the high hole density of graphene (i.e., OH^-^ enhances P-doping). The study estimated that the detection limit was 0.025. Ang et al. studied the sensing behavior of solution-gated graphene FET to pH using FLG prepared through epitaxial growth (Figure 8b) [118]. The mobility of the charge carriers in 3–4 layered graphene is about 15% higher than that in 1–2 layered graphene. The FLG samples were fabricated by annealing n-type H-SiC surface at 1100 °C, accordingly, graphene prepared by epitaxial growth was n-doped. The conductive curve of 1–2 layered graphene shows a larger shift to a negative gate voltage than the 3–4 layered graphene because the 1–2 layered graphene had a stronger coupling effect with the electron-doped substrate. The increase in pH also led to a shift to the right of the Dirac point, which indicated the increased p-doping caused by adsorption of OH^-^. This result is inconsistent with Ohno et al.’s, though their graphene was mechanically exfoliated and p-doped. The change in the threshold voltage of the Dirac point shift caused by the per unit pH change for both 1–2 layered (99 mV/pH) and 3–4 (98 mv/pH) layered graphene was observed to be larger than the theoretical Nernst limit when the negative gate voltage was applied. The over limit response of FLG to pH in the negative gated potential region suggests the potential of solution-gated graphene FET as an ultra-sensitive biosensor and chemical sensor. Mailly-Giacchetti et al. studied how resist residues affect the pH sensing behavior of a graphene solution-gated FET sensor [119]. Graphene was prepared by CVD and was transferred onto a Si/SiO2 substrate where the source and drain electrodes (Ti/Pt/Au) were then prepared by EBL. To fabricate a patterned graphene channel, MMA was spin-coated on graphene to serve as a protective layer before spin-coating the photoresist layer. The devices were then subject to O_2_ plasma etching and rinsed with acetone to remove the photoresist and MMA layers. Thermal annealing (500 under H_2_/Ar flow) was applied to fabricate devices with completely cleaned graphene surfaces. Tests were also conducted on devices without thermal annealing treatment for comparison. The device with the clean graphene surface was found to have a more significant p-doping effect than the device with a residue-on-top graphene surface and, according to authors, the stronger p-doping was caused by high-temperature annealing. However, the increase in per unit pH led to a similar shift to the right of the Dirac point for the device with a completely cleaned graphene surface (22 mV/pH) and the device with a residue-on-top graphene surface (21 mV/pH). The authors also studied whether pH sensing would be affected if the solid Si/SiO_2_ substrate was replaced by a flexible poly (ethylene 2,6-naphthalene dicarboxylate) (PEN) substrate. The device with the PEN substrate had less p-doping for the lack of annealing. It had lower transconductance because of rough PEN surface caused lower carrier mobility. Whereas, the PEN substrate showed no impact on the sensitivity of graphene FET to pH, suggesting a proof-of-concept wearable pH sensor based on graphene FET (Figure 8c). The research further examined whether surface doping would impact pH sensing by fabricating the device on octadecyl trichlorosilane (OTS). As a result, graphene was slightly n-doped. However, the response of the device to pH was still not affected. Sudibya et al. developed an rGO FET that can detect metal ions [120]. A GO aqueous solution was drop-cast on a quartz substrate modified by APTES and was reduced by hydrazine vapor. After the device was cleaned by thermal annealing, a PASE linker was used to immobilized calmodulin (CaM) which is a Ca^2+^ protein to rGO for the detection of Ca^2+^ and Mg^2+^. The adsorption of Ca^2+^ and Mg^2+^, equivalent to positive-potential gating, led to a decrease in the conductance. To achieve heavy metal detection, the rGO surface was functionalized with a metallothionein type II protein (MT-II) with the aid of a PASE liner. The device could then detect Hg^2+^ and Cd^2+^ down to 1 nM. However, Hg^2+^ and Cd^2+^ increased the conductivity. As the isoelectric point of it is 3.85, MT-II is negatively charged in a neutral solution. Binding of metal ions significantly changed the conformation of the negatively charged MT-II, making it closer to the rGO surface and thus strengthening negative-potential gating effect. Moreover, as the MT-II immobilization resulted in the p-doping effect, the conformational change of MT-II might further enhance the p-doing effect and thus increase the conductivity. The device was reusable after being washed in acidic buffer. Park et al. fabricated a flexible graphene-based FET integrated with human olfactory receptors as a bioelectronic nose [121] (Figure 8f). Bilayer graphene was prepared by CVD and was transferred to a flexible poly (ethylene terephthalate) (PET) substrate decorated with gold electrodes. P-type graphene (OG) and n-type graphene (NG) were prepared by treatment with oxygen plasma and ammonia plasma, respectively, followed by thermal annealing to restore the physical and electrical properties of graphene. Human olfactory receptor (OR), which selectively binds to odorant amyl butyrate (AB) molecules, was immobilized to the graphene surface via a diaminonaphthalene (DAN) agent. DAN can conjugate to graphene through π–π stacking. Meanwhile, it can covalently bind to OR using GA as a linker. The transfer curves for both OG and NG showed a shift to the right upon the adsorption of AB. OR belongs to the G-protein-coupled receptor family that usually exhibits a conformational equilibrium between an uncharged inactive and a negatively charged active state due to the switching between the RSH and the negatively charged RS^-^ of the thiol group of cysteine. The structure of OR can be rearranged to a negatively charged state upon binding to AB, which causes the negative-potential gating effect. Thus, a high hole density increases the conductivity of OG but a decrease of NG (Figure 8g). The sensitivities of OG and NG were compared by the authors, and OG was found to be more sensitive with a detection limit down to 0.04 fM. The sensor was flexible and stable. It maintained good performance after 100 cycles of bending and relaxing (Figure 8h).

### 4.4. Acoustic, Strain, Magnetic, and other Mechanical Sensors

**Figure 9:**
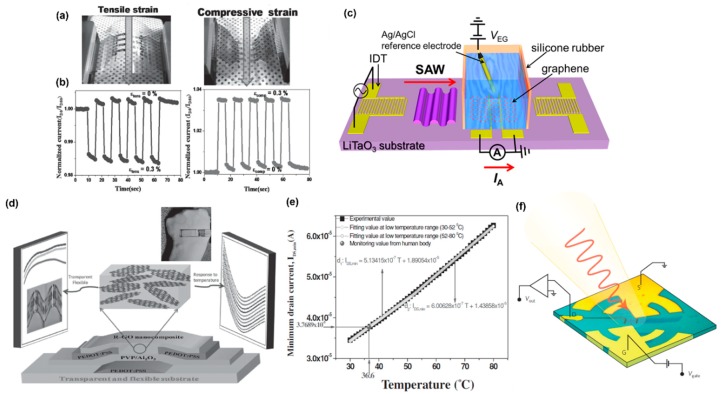
(**a**) Image of rGO FET subject to compressive and tensile strain. (**b**) The response of $I_{d}$ of the rGO FET strain sensor to compressive and tensile strain. (**c**) Design of graphene FET SAW sensor. (**d**) Flexible and wearable rGO/P(VDF- TrFE) nanocomposite temperature sensor. Insert: Photograph of the sensor worn on human skin. (**e**) Measurement of body temperature shown by $I_{d}$. (**f**) Graphene FET terahertz detector. Reprinted with permission from References [122,124,130,131].

Trung et al. developed an ultrasensitive and wearable strain sensor using rGO FET (Figure 9a) [122]. A poly-4-vinyl phenol (PVP) dielectric layer was spin-coated onto a gate electrode (Ni) deposited flexible polyethersulfone substrate (PES). A 20 nm thick Al_2_O_3_ buffer layer was then deposited on the PVP layer and worked as the substrate for the printed poly (diallyldimethylammonium chloride) (PDDA) pattern on which GO would selectively bind. GO was then reduced by hydrazine vapor. After that, the Au/Cr source and drain electrodes were decorated onto the substrate. To avoid the p-doping effect caused by oxygen and water in the environment, the whole device was subjected to annealing and was then encapsulated by a tetratetracontane layer. Tensile strain led to a decrease in $I_{d}$, while compressive strain led to an increase in $I_{d}$ (Figure 9b). The device possessed electromechanical stability. The response signal of it was still reliable after 10,000 bending cycles. Miseikis et al. demonstrated that graphene on a piezoelectric substrate (lithium niobate) exhibited an acoustoelectric effect, which was the generation of current in a low-dimensional system due to surface acoustic waves (SAW) [123]. To exploit this property of graphene, Okuda et al. developed a SAW sensor using a graphene FET design, which could achieve simultaneous detection of charge and mass (Figure 9c) [124]. Graphene was transferred onto the middle of a source/drain electrode decorated Y-cut LiTaO3 substrate, which was piezoelectric. The graphene FET was surrounded by a silicon rubber well. Two pairs of interdigital transducers (IDT), which were composed of Ni/Au, were fabricated outside the well opposite each other. SAW, thus, was generated when a high-frequency signal input was applied to the IDT. The well was filled with a phthalate buffer solution (pH 4.1) and an Ag/AgCl reference electrode was immersed in the buffer through which the electrolyte-gate ($V_{Eg}$) was applied. Nonmagnetic silica microbeads, which were modified by amine groups, were dropped into the buffer. A shift to the left of the $I_{d}$-$V_{Eg}$ curve and a downward shift of the acoustoelectric current ($I_{A}$)-$V_{Eg}$ curve was observed. The addition of amine groups carried by the microbeads into the buffer was equivalent to positive potential gating, leading to the left shift of the $I_{d}$-$V_{Eg}$ curve. Meanwhile, the microbeads increased the mass loading onto the propagation path of SAW, resulting in a decrease in the amplitude of SAW. Thus, charge and mass could be simultaneously detected. The authors also demonstrated the usage of the device as a conventional pH sensor. The magnetoresistance effect (MR) is the change in resistance due to the application of an external magnetic field. The MR on a material is either dominated by a physical contribution of material’s parameters or a geometric contribution, which depends on a circuit path, sample pattern, and the electrode configuration [125,126]. The extraordinary MR (EMR is the significant increase in MR due to the geometric contribution, which is orders of magnitude stronger than MR due to the physical contribution) was found by Solin et al. in a patterned high-mobility and narrow-gaped semiconductor (InSb) [125]. An EMR device usually comprises of a high-mobility semiconductor and a metal shun that is connected to the semiconductor and is modulated by the Lorentz force, caused by current deflection between two conduction channels, which are formed between semiconductors and metal shunts [127]. High charge carrier mobility, the one-atom-thick sensing layer, and durability to the high current density of graphene, make graphene a prospective material for use as a magnetic field sensor at a high spatial resolution [128,129]. Pisana et al. investigated a graphene-metal EMR device [129]. Graphene was obtained through exfoliating highly oriented pyrolytic graphite and transferred onto a Si/SiO_2_ substrate. Ta/Au electrodes and shunt were fabricated through EBL and lift-off. The device was able to detect magnetic fields at the nanoscale with a tunable signal to noise ratio. Trung et al. fabricated a transparent and flexible temperature sensor using a nanocomposite composed of rGO and P(VDF-TrFE) as a channel for FET (Figure 9d) [130]. GO and P(VDF-TrFE) were mixed in DMAC to form the composite and then GO was reduced by hydrazine vapor and thermally annealed. PEDOT:PSS was spin-coated on a PES substrate working as the back gate. Al_2_O_3_/PVP/Al_2_O_3_ was used as the dielectric layer. PEDOT:PSS was then spin-coated on the dielectric layer and was patterned to be the source/drain electrodes. The RGO/ P(VDF-TrFE) nanocomposite was then spin-coated on the top. The sensor was effective within a large temperature range (30–80 °C) and the detection limit of the sensor was 0.1 °C. The sensor was able to successfully capture human body temperature while attached to human skin (Figure 9e). The flexibility of a wearable sensor enabled it to maintain good performance after 10,000 bending cycles. Graphene, by virtue of its high carrier mobility and frequency-independent adsorption, is a prospective material that operates in the terahertz region. Vicarelli et al. developed an antenna-coupled graphene FET terahertz detector (Figure 9f) [131]. Mechanically exfoliated SLG and BLG were transferred to a Si/SiO_2_ substrate. A patterned source electrode in the shape of a long-periodic circular-toothed antenna and drain electrode ribbon were fabricated by EBL and lift-off. An atomic thick HfO_2_ layer was deposited as the dielectric gate. A Cr/Au antenna with the same pattern as the source electrode was then fabricated on top; working as the top gate. A BLG detector showed a linear response to terahertz power. BLG exhibited a higher conductivity and sensitivity (~30 nW Hz^−1/2^) one order of magnitude higher than SLG.

## 5. Electrochemical Sensor 

Electrochemical impedance spectroscopy (EIS) is a technique to study ionic conductivity and investigate the electrochemical behavior of materials by applying an alternating current with changing frequency. Electrical features of a system are subject to the surface phenomenon in an EIS test. Detection of binding activity on the electrode surface using electrochemical impedance spectroscopy (EIS) has been widely applied in the field of biosensors [132]. The large specific surface area due to the atomic-thick 2D structure of graphene and the conjugated π system enabled fast charge carrier mobility and makes graphene a promising electrocatalytic transduction material at the electrode/electrolyte interface. Besides, GFM subject to facile and low-cost printing techniques such as inkjet, screen, direct-write printing, are emerging as material for flexible electrodes in wearable sensors. [133] 

### 5.1. Electrochemical Sensor Based on GFM

**Figure 10:**
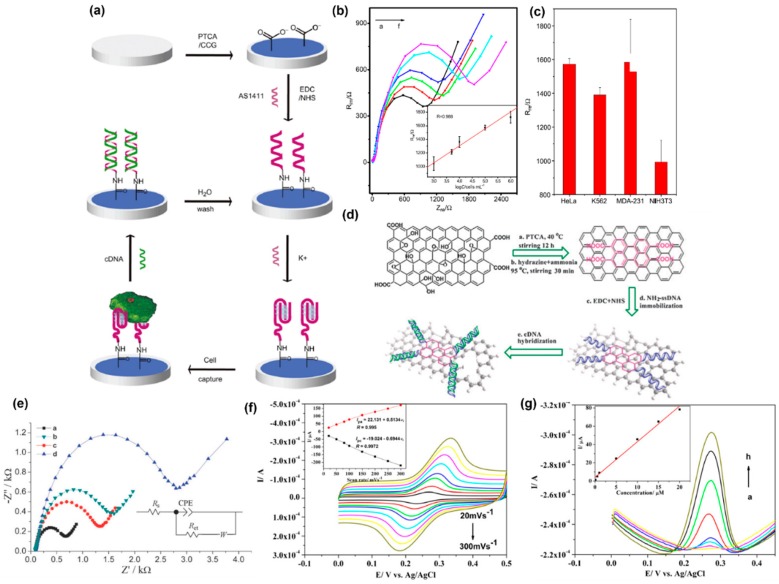
(**a**) Reusable AS1411/graphene electrochemical aptasensor. (**b**) Electrochemical impedance spectroscopy (EIS) Nyquist plot of the PTCA/CCG modified GCE exposed to HeLa cells which is a cancer cell line at different concentrations (cell/mL): a. 0, b. 1 $\times 10^{3}$, c. 5 $\times 10^{3}$, d.1 $\times 10^{4}$, e. 1 $\times 10^{5}$, f. 1 $\times 10^{6}$. Insert: R_et_ increases linearly with the logarithm of cell concentration. (**c**) R_et_ value of PTCA/CCG modified GCE immersed in a suspension of three cancer cell lines (HeLa, K562, and MDA-231) and a normal cell line (NIH3T3). (**d**) Scheme of the immobilization of NH_3_-ssDNA to PTCA/rGO and binding of the target DNA. (**e**) Nyquist plot of GCEs: a. bare, b. GO-coated, c. graphene-coated, d. PTCA/rGO-coated. (**f**) CV of rGO-coated GCE in the presence of paracetamol in NH_3_·H_2_O–NH_4_Cl buffer at different scan rates. (**g**) Square-wave voltammetry (SWV) of rGO-coated GCE showing the response of $I_{ap}$ to paracetamol at different concentrations. Reprinted with permission from References [98,134,135].

Qu’s group fabricated a label-free electrochemical aptasensor on a graphene platform that can selectively distinguish cancer cells from normal ones and the detection limit was down to $1\times 10^{3}$ cells/mL (Figure 10a) [10,134]. Nucleolin, an overexpressed protein on the plasma membrane of tumor cells, was the target of their sensor. AS1411, a 26-mer DNA aptamer with a high affinity to nucleolin, has been used for the treatment of myeloid leukemia and renal cell carcinoma in phase II clinical trials. To enhance graphene dispersibility and to increase negatively charged OCGs of GO, 3,4,9,10-perylene tetracarboxylic acid (PTCA), that can anchor to graphene basal plane through π–π stacking and hydrophobic interaction, was employed in the reduction process of GO and formed PTCA-functionalized graphene (PTCA/graphene). NH_2_-modified AS1411 was anchored to PTCA/graphene via EDC/NHS coupling. AS1411 anchored PTCA/graphene was immobilized on a glassy carbon electrode (GCE) via nafion. AS1411 could capture cancer cells via forming a stable G-quadruplex structure with nucleolin on the cell surface, leading to an increase in the electron transfer resistance (R_et_), which can be determined by the semicircle diameter (Figure 10b). Additionally, the increase of R_et_ was in proportion to the increase in cancer cell concentration on a logarithmic scale (Figure 10). R_et_ only increased significantly when the PTCA/graphene modified GCE was exposed to cancer cell lines (Figure 10c), demonstrating the strong selectivity to cancer cell detection of the sensor. Complementary DNA (cDNA) of AS1411, which can hybridize the aptamer and compete for the binding site with nucleolin, can be used to detach the captured cancer cells. The affinity of AS1411 to nucleolin can be restored after 30 s at room temperature by distilled water. In that way, a reusable electrode for cancer cell detection was achieved. Similarly, Hu et al. developed a label-free DNA sensor using PTCA/rGO-coated GCE and NH_2_-modified ssDNA was anchored to work as a probe (Figure 10d) [98]. R_et_ of bare GCE, as well as GCE coated by GO, rGO, and PTCA/rGO, were studied using [Fe(CN)6]^3−/4−^ redox coupling (Figure 10e). R_et_ of GO-coated GCE was notably larger than the other three, indicating an insulating effect of GO due to the disrupted conjugated π system by OCG. However, R_et_ of PTCA/rGO-coated GCE was slightly increased compared to graphene-coated GCE, suggesting that PTCA had the virtue of both a high conductivity and chemically active OCG. Thus, PTCA offered a way to introduce -COOH groups to graphene without making sacrifices in conductivity. The binding of target DNA caused an increase in R_et_. It has been explained by the authors that the formation of a more negatively charged dsDNA increased the electrostatic repulsion to [Fe(CN)6]^3−/4-^ and lifted the DNA structure, making it harder for [Fe(CN)6]^3−/4-^ to interact with GCE. GFM coated GCEs can also work as sensors for other chemical molecules besides a biosensor. Kang et al. designed an electrochemical sensor for paracetamol detection by exploiting the electroactive properties of paracetamol and rGO-coated GCE [135]. All measurements were conducted in an NH_3_·H_2_O–NH_4_Cl buffer. Cyclic voltammetry (CV) was performed at different scan rates for rGO-coated GCE in the presence of paracetamol. Well-defined redox peaks at different scan rates were exhibited (Figure 10f). Besides, cathodic and anodic peak currents exhibited a linear relationship with the scan rate. Due to the interaction that caused a strengthened adsorption and high carrier mobility of rGO, rGO can offer sufficient electrons for the reduction process in the redox reaction of paracetamol quickly, making the redox reaction quasi-irreversible at the rGO interface. Besides, defects in rGO provide electrocatalytic sites for redox reactions. Square-wave voltammetry (SWV) was used for the detection of paracetamol and the anodic peak current ($I_{ap}$) increased linearly with the concentration of paracetamol (Figure 10g). 

### 5.2. Electrochemical Sensor Based on GFM Composite

**Figure 11:**
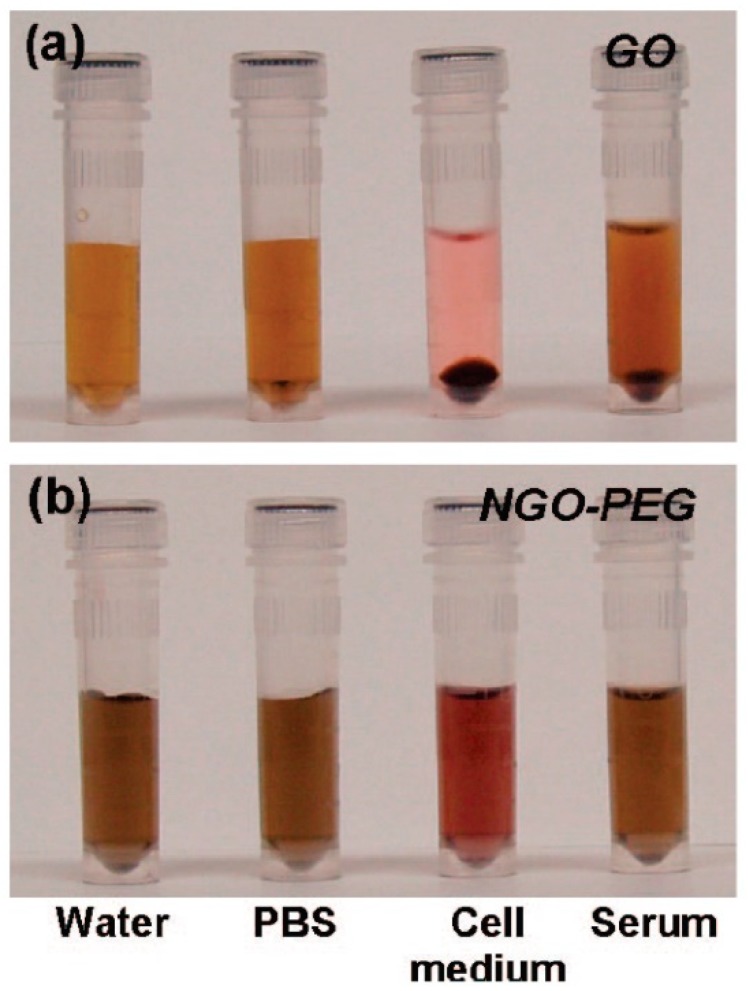
Photos of (**a**) GO and (**b**) NGO-PEG solutions that dissolved against different solvents. Reprinted with permission from References [137].

Metal nanoparticles such as AuNP can help with the immobilization of biomolecules to GFM sheets and provide a suitable microenvironment that maintains a biomolecules’ biological activity. Besides, some metal nanoparticles (e.g., PtNP) can work as electrocatalytic sites for analytes (e.g., H_2_O_2_) [136]. Chitosan, which is discussed above as a coating for GO sheets to decrease cytotoxicity, is also widely used as a protective coating for electrodes of electrochemical sensors due to its excellent film-forming ability, good biocompatibility, outstanding permeability. Polyethylene glycol (PEG) is a highly branched biocompatible polymer that can form a composite with GO through carbodiimide intermediated coupling, and the composite (NGO-PEG) shows higher solubility and stability in various kinds of solutions than GO (Figure 11) [137]. Thus, forming a composite with nanoparticles or/and biocompatible polymers can extend the application of GFM in biosensing. Jin et al. incorporated an rGO/Pt composite-based electrochemical sensor into microneedles (MNs) and achieved transdermal H_2_O_2_ sensing in vivo [138]. Pt NP, as a metal nanoparticle, can bind to graphene non-covalently through strong van der Waal interactions to bind to rGO through the remaining OCG [136]. To fabricate rGO/Pt composite decorated MNs, MNs were immersed into a mixture of H_2_PtCl_6_ and GO aqueous solution, which was sonicated ahead. After the coating was dry, MNs were immersed in Vitamin C to simultaneously reduce GO and H_2_PtCl_6_. Thus, Pt NPs were able to be deposited onto the rGO surface. To avoid damage to the rGO/Pt coating during the insertion of MNs into the skin, a protective polyvinylpyrrolidone (PVP) layer was spray-coated onto the rGO/Pt-decorated MNs. To achieve real-time and in situ transdermal biosensing, three MN patches with different decoration coatings were assembled into the three-electrode system: a Pt/rGO MN patch acting as a working electrode, an MN patch sputter-coated Pt as the counter electrode, and an MN patch dip-coated with Ag/AgCl as the reference electrode. The sensor detected the H_2_O_2_ level of the different concentrations of H_2_O_2_ soaked pig skins and gave out a signal when mice were injected with H_2_O_2_, manifesting successful transdermal H_2_O_2_ sensing both in vitro and in vivo. Wang et al. adopted chitosan in an rGO-based composite H_2_O_2_ sensor to enhance the attachment of the rGO-based composite on GCE [139]. Hemin is the electrocatalytic species that was incorporated into the composite. AuNPs worked as the core for the composite to enhance the attachment of Hemin (Hem) and to improve electrocatalytic activity. AuNPs and hemin formed the Hem@AuNP composite through gold biomineralization. Then, rGO was mixed with the composite to form the Hem@AuNP/rGO composite. Finally, the composite was mixed with chitosan. Halder et al. used polyethylenimine (PEI), which is also a highly branched polymer with an amine group to reduce GO and it can simultaneously form a biocompatible scaffold around rGO [140]. Ferrocene carboxylic acid (FCA), the ferrocene moiety which is a good electron acceptor for enzymes [141], was then incorporated to the scaffold through an EDC/Hobt intermediated coupling reaction. After that, the as-prepared composite was mixed in an ethanolic Nafion solution and drop-cast onto GCE. Different enzymes (e.g., GOD and Cholesterol oxidase) then can be drop cast on the modified electrodes. Thus, the detection of different analytes can be achieved. The sensor showed A fast response, a good reusability, and a high sensitivity. The sensor successfully measured glucose and cholesterol levels in serum samples obtained from a human.

## 6. Conclusions and Future Directions

The electrical, mechanical, optical, thermal, and chemical properties of GFM members—GO, rGO, and graphene—were compared in this review. Besides, causes for differences in properties were also discussed. Biocompatibility and interaction with biomolecules were summarized. The 2D surface of GFM itself contains binding sites for ssDNA due to the strong affinity. Besides, OCG of GO and rGO, especially, the -COOH groups, are active sits for aptamer immobilization via chemical modification such as carbodiimide intermediated coupling. Thus, as an effective quencher for fluorescence due to the high FRET rate, GFMs are promising materials for fluorescence-based apasensors. The atomic thickness, unique Hall effect, and acting as p-doped semiconductor due to air exposure make GFMs ideal materials for FET. Adsorption of gas molecules imposes doping effects on GFMs, which leads to changes in FET conductance and makes GFM FET an ultrasensitive gas sensor. The carboxyl groups of GO facilitate stronger anchoring to amine-group modified substrates through electrostatic interactions. Due to the conjugated π system, PASE linkers and PTCA can be used to immobilize bio-probes without damaging conductivity. Binding of biomolecules to bio-probes on GFM results in a gating effect and thus changes in the conductivity of GFM, which is the principle for the GFM FET biosensor. Annealing is usually employed to create a clean surface for the further immobilization of bio-probes. However, annealing increases the conductivity of the FET gas sensor at the expense of sensitivity. In addition, FET on a flexible substrate sheds light on the design for wearable sensors. Moreover, using GFM as the interface for the electrochemical sensor due to the electrochemical active defects and fast electron transportability has been illustrated in this review.

GFMs with a large aspect ratio are intriguing nanofillers for composite materials. On the one hand, GFMs can enhance the mechanical and electrical properties of them, meanwhile, GFMs can bring an ultra-sensitivity that can respond to various stimuli. On the other hand, other components of the composite can improve the properties of GFMs (e.g., cytotoxicity) and the synergistic effects of the cooperation of all components might bestow the composite with novel properties. Studies mainly elucidate the application and assembly of GFM composite materials. However, the underlying mechanism for property enhancement and development is yet to be studied.
